# A Cystine-Cysteine Intercellular Shuttle Prevents Ferroptosis in xCT^KO^ Pancreatic Ductal Adenocarcinoma Cells

**DOI:** 10.3390/cancers13061434

**Published:** 2021-03-21

**Authors:** Willian Meira, Boutaina Daher, Scott Kenneth Parks, Yann Cormerais, Jerome Durivault, Eric Tambutte, Jacques Pouyssegur, Milica Vučetić

**Affiliations:** 1 Department of Medical Biology, Centre Scientifique de Monaco (CSM), 98000 Monaco, Monaco; wmeira@centrescientifique.mc (W.M.); bdaher@centrescientifique.mc (B.D.); jdurivault@centrescientifique.mc (J.D.); 2 Trev and Joyce Deeley Research Centre, BC Cancer, Victoria, BC V8R 6V5, Canada; sparks@bccrc.ca; 3 Genome British Columbia Proteomics Centre, University of Victoria, Victoria, BC V8Z 7X8, Canada; 4 Department of Molecular Metabolism, Harvard T.H. Chan School of Public Health, Boston, MA 02115, USA; ycormerais@hsph.harvard.edu; 5 Department of Marine Biology, Centre Scientifique de Monaco (CSM), 98000 Monaco, Monaco; etambutte@centrescientifique.mc; 6 CNRS, INSERM, Centre A. Lacassagne, Institute for Research on Cancer & Aging (IRCAN), University Côte d’Azur, 06107 Nice, France

**Keywords:** cysteine-cystine shuttle, ferroptosis, resistance, tumor environment, cysteine transporters

## Abstract

**Simple Summary:**

The xCT transporter of oxidized form of cysteine has been recognized as fundamental for cellular amino acid and redox homeostasis. Increasing number of data suggests that xCT inhibition-induced ferroptosis has great potential for development of novel anti-cancer therapeutics for pancreatic cancer patients. The aim of this study was to investigate potential resistance mechanisms that cancer cells with genetically disrupted xCT (xCT^KO^) may exploit in order to develop resistance to ferroptosis. Our data clearly showed that shuttle of reduced cysteine between cancer xCT^KO^ and neighboring cells provide protection of the former. Importantly, this shuttle seems to be fueled by the import and reduction of oxidized cysteine by xCT-proficient feeder layer. In summary, two important findings are: (1) supply of the reduced cysteine has to be taken in consideration when xCT-based ferroptosis inducers are used, and (2) systemic inhibition of xCT could be potential approach in overcoming this resistant mechanism.

**Abstract:**

In our previous study, we showed that a cystine transporter (xCT) plays a pivotal role in ferroptosis of pancreatic ductal adenocarcinoma (PDAC) cells in vitro. However, in vivo xCT^KO^ cells grew normally indicating that a mechanism exists to drastically suppress the ferroptotic phenotype. We hypothesized that plasma and neighboring cells within the tumor mass provide a source of cysteine to confer full ferroptosis resistance to xCT^KO^ PDAC cells. To evaluate this hypothesis, we (co-) cultured xCT^KO^ PDAC cells with different xCT-proficient cells or with their conditioned media. Our data unequivocally showed that the presence of a cysteine/cystine shuttle between neighboring cells is the mechanism that provides redox and nutrient balance, and thus ferroptotic resistance in xCT^KO^ cells. Interestingly, although a glutathione shuttle between cells represents a good alternative hypothesis as a “rescue-mechanism”, our data clearly demonstrated that the xCT^KO^ phenotype is suppressed even with conditioned media from cells lacking the glutathione biosynthesis enzyme. Furthermore, we demonstrated that prevention of lipid hydroperoxide accumulation in vivo is mediated by import of cysteine into xCT^KO^ cells via several genetically and pharmacologically identified transporters (ASCT1, ASCT2, LAT1, SNATs). Collectively, these data highlight the importance of the tumor environment in the ferroptosis sensitivity of cancer cells.

## 1. Introduction

Despite great efforts in the development of new diagnostics and treatments, cancer is still a major threat for human health with rising incidence and mortality rates [1,2]. Targeting cell death is one of the most common strategies in anti-cancer treatment. For decades, the idea of reprogramming intrinsic cell death pathways to fight tumor cells has focused on apoptosis [3,4]. However, concomitantly, additional studies showed that resistance to apoptotic cell death induction is one of the hallmarks of cancers, frequently associated with overexpression of anti-apoptotic proteins such as BCL-2 or by the loss of function of tumor suppressor TP53 [5].

In 2012, Dixon et al. [6] described a new type of cell death called ferroptosis, shedding light on a completely new and highly promising pathway for anti-cancer therapy research. Ferroptosis is a regulated, iron-dependent type of cell death characterized by the accumulation of lipid hydroperoxides (LOOH) in response to oxidative stress and oxidation of membrane lipids, mostly polyunsaturated fatty acids (PUFAs), which compromises the permeability and fluidity of the membrane lipid bilayer, eventually leading to cell death [6]. Under basal conditions cells are protected from this uncontrolled membrane damage by the presence of the specific selenoenzyme glutathione peroxidase 4 (GPx4), which plays a critical role in reducing membrane lipid hydroperoxides (LOOH) into non-toxic lipid alcohols (LOH) using glutathione (GSH) as a co-substrate reducing agent [7]. Indeed, impaired GPx4 activity induced by either direct inhibition or decreased intracellular levels of its reducing power, GSH has been recognized as a cause of the accumulation of lipid hydroperoxides and ferroptotic cell death [8]. Intracellular level of GSH depends on the import of cysteine in its oxidized (dominant) form (also known as cystine, CySSCy), through the system xc^-^. This transport system, consisting of the substrate-specific subunit named xCT (SLC7A11) and the chaperone CD98 (SLC3A2), promotes the import of CySSCy at the expense of glutamate (Glu) efflux in a Na^+^-independent way [6,9,10].

In our previous study [10], we showed that genetic disruption of the xCT transporter in pancreatic ductal adenocarcinoma (PDAC), one of the most aggressive types of cancer with a dismal 5-year survival rate of 7% [11], promotes intracellular cysteine depletion, inhibition of GSH synthesis followed by accumulation of membrane lipid hydroperoxides and finally, ferroptotic cell death in vitro. Similar results have been observed with pharmacological inhibition of xCT by erastin in a variety of cancers including breast, liver, gastric and pancreatic tumors [12,13,14,15]. However, in the same study our group demonstrated that, contrasting with the in vitro results, xCT-depleted PDAC cell xenografts in mice were able to grow at the same rate as their wild type (WT) counterparts, albeit with a slight delay. Additionally, we observed the presence of infiltrated fibroblasts within the tumor mass, suggesting that these cells may be involved in an adaptive mechanism of the xCT^KO^ cells in vivo [10]. Corroborating with our results, a study published the same year by Arensman et al. [16] showed similar results demonstrating that adenocarcinoma MC38 xCT-depleted cells exhibited inhibition of proliferation in vitro, but in vivo were able to develop tumors, also with a delay.

Considering our previous observations [10] and in order to understand the resistance to ferroptotic cell death of xCT^KO^ cells in vivo, this study aimed to investigate the role of adjacent fibroblasts in the resistance of xCT^KO^ PDAC cells, while also identifying the importance of the amino acid transporters involved in the interplay between xCT-expressing cells and xCT-disrupted cells. Here we report that any cell type expressing xCT are able to provide a supply of cysteine (CySH) for xCT-disrupted tumor cells, consequently conferring a resistance to ferroptosis cell death. We demonstrated the synergistic activity of different CySH transporters, such as ASCTs, SNATs and LAT1 suggesting that although impairment of CySH homeostasis appears to be a good strategy to induce ferroptotic cell death in PDAC tumors, the complexity of intercellular metabolic cooperation should be contemplated seriously in order to overcome resistance.

## 2. Results

### 2.1. Fibroblasts Are Capable of Reversing the Ferroptotic Phenotype of xCT^KO^ PDAC Cells

**Figure 1:**
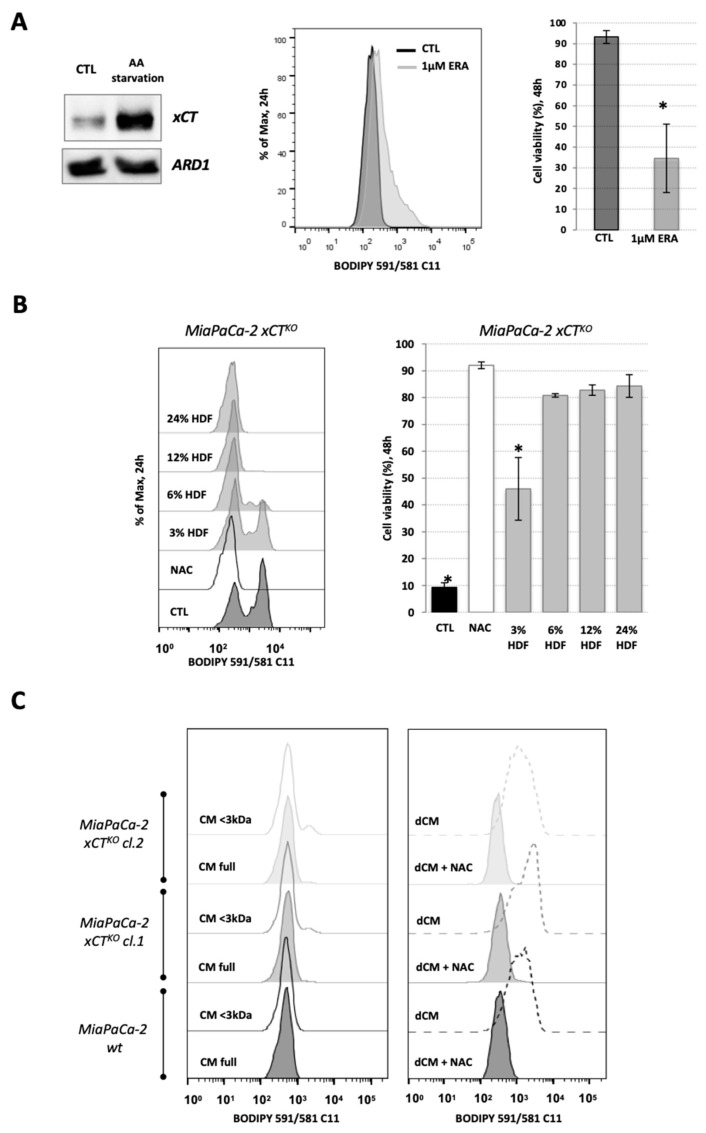
Fibroblasts reverse the characteristic ferroptosis phenotype of pancreatic ductal adenocarcinoma (PDAC) xCT^KO^ cells. (**A**). The expression level of xCT in human dermal fibroblasts (HDF) under basal and amino acid (AA) starvation conditions (Western blot, left panel—ARD1 used as loading control), their sensitivity to xCT inhibition by erastin (ERA) seen through the lipid hydroperoxide accumulation after 24 h (BODIPY 591/581 C11 staining, middle panel), i.e., cell viability after 48 h (right bar graph). (**B**)**.** Optimization of the co-culture conditions: accumulation of the lipid hydroperoxides (left) and cell viability (right) of MiaPaCa-2 xCT^KO^ cells co-cultured with 3%, 6%, 12% or 25%, of HDF cells during 24 h and 48 h, respectively. (**C**)**.** BODIPY 591/581 C11 staining of MiaPaCa-2 wild type (wt) and xCT^KO^ cells cultured for 24 h in full or fractionated (lower (<) or higher (>) then 3 kDa, left and right panel, respectively) conditional media (CM) of HDF; dCM = dialyzed conditional media (>3 kDa). All experiments have been performed in triplicate and the representative blots and histograms are shown. Bar graphs show mean ± SEM; n = 3; *, *p* < 0.05, comparison with control group.

In order to understand the role of neighboring cells in sustaining the in vivo survival of genetic-disrupted xCT PDAC cells as suggested by Daher et al. [10], human dermal fibroblasts (HDF) and xCT-disrupted MiaPaCa-2 cell lines (previously described, [10]) were used to address this issue in vitro. The expression of xCT in HDF cells was assessed by Western blot in control conditions and amino acid starvation conditions, so that maximal expression of the xCT is detected (Figure 1A). Under AA-stress, the GCN2-ATF4 pathway is activated, promoting the expression of ATF4-targeted genes, including xCT [17], as shown via Western blot in HDF cells (Figure 1A).

Additionally, the response of HDF cells to ferroptosis induction was evaluated. Ferroptosis is characterized by an accumulation of membrane lipid hydroperoxides that leads to cell death and can be triggered by depletion of cystine/cysteine (CySSCy/CySH) uptake and, consequently, decrease in glutathione (GSH) synthesis [6]. Thus, erastin was used, as it binds irreversibly to xCT transporters and inhibits the uptake of cystine (CySSCy), the main form of cellular cysteine import [18]. FACS analysis showed that HDF cells are sensitive to 1 μM erastin (Figure 1A), as shown by the lipid hydroperoxide accumulation after 24 h of incubation, measured by BODIPY 591/581 C11 fluorescent staining, and an approximately 70% decrease in cell viability after 48 h, as measured by the DNA staining of dead cells by propidium iodide (PI) (Figure 1A).

Furthermore, the capacity of HDF cells (hereafter also referred to as “host cells”) to reverse the ferroptotic phenotype of MiaPaCa-2 xCT^KO^ (hereafter also referred to as “guest cells”) was assessed by co-culture of these two cell lines in different ratios (3%/97%, 6%/94%, 12%/88%, and 25%/75%, host-to-guest cells). The major reason for decreasing the number of “host” cells was that they were indistinguishable from the “guest” cells in the FACS analysis (see FSC/SSC plot, additional data file) and in order to avoid the signal coming from fibroblasts to overwrite the signal coming from xCT^KO^ cells. As shown previously [10], when seeded without the alternative cysteine donor N-acetylcysteine (NAC), MiaPaCa-2 xCT^KO^ cells exhibited increased accumulation of membrane lipid hydroperoxides after 24 h and a 90% decrease in cell viability by 48 h (Figure 1B). Figure 1B shows that this characteristic phenotype of xCT^KO^ cells was partially reversed in the co-culture with 3% of the “host cells” and completely prevented at higher ratios. Therefore, the co-culture with 6% of host cells was defined as the standard for future assays. It is worth noting that the host cells, in each analysis, have been pre-seeded 24 h before the guest cells.

In order to identify if this rescue effect was due to the cell-to-cell contact or the guest cells export of the “rescue agent”, MiaPaCa-2 xCT^KO^ cells were cultivated in the conditioned media (CM) from HDF cells and the level of lipid hydroperoxides was assessed. Full conditioned media from HDF cells had no effect on MiaPaCa-2 wt cells whereas it completely prevented the ferroptosis phenotype of MiaPaCa-2 xCT^KO^ (two independent clones; Figure 1C). Following this, the CM from HDF cells was fractionalized by centrifugation with 3 kDa cut-off filters and no lipid hydroperoxide accumulation was observed in the xCT^KO^ cells cultivated with the CM fraction that only contains components which have a MW that is less than 3 kDa (<3 kDa, Figure 1C). However, it is important to point out that a significant concentration of phenol red (354.38 Da) remained after the centrifugation, indicating the presence of less than 3 kDa molecules in the >3 kDa fraction. In order to overcome this issue, the fraction containing only components higher than 3 kDa was obtained by dialyzing the CM against PBS buffer using a 3 kDa cut-off membrane (Spectrum Laboratories, Inc, Waltham, MA, USA). Cultivation of both, MiaPaCa wt and xCT^KO^ cells, in dialyzed CM (dCM) lead to increased membrane lipid hydroperoxides that was reverted to control levels by the addition of NAC (Figure 1C), suggesting that guest cells secrete the “saving agent”, which is less than 3 kDa and that is then taken up by the neighboring xCT^KO^ cells to be utilized for the prevention of ferroptosis.

### 2.2. xCT-Expressing Cells Export a Redox-Sensitive Agent Which Prevents Ferroptosis and Restores Amino Acid Balance in xCT^KO^ Cells

**Figure 2:**
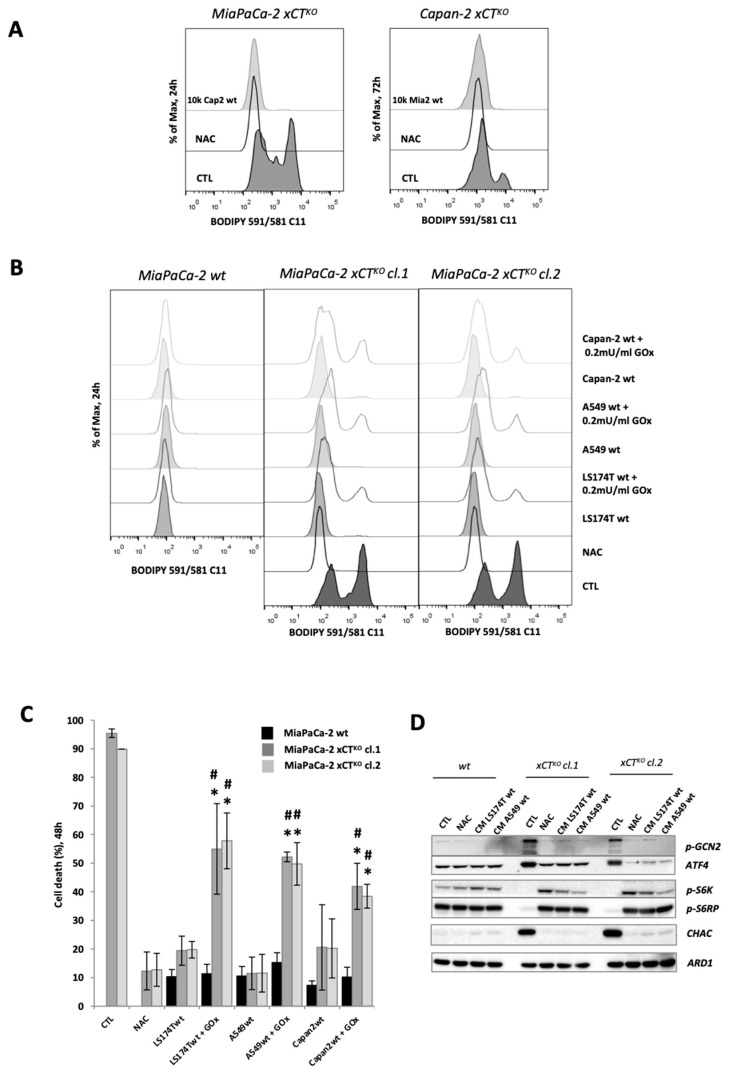
xCT-expressing cells export a redox-sensitive agent which prevents ferroptosis and restores amino acid imbalance in xCT^KO^ cells. (**A**). Lipid hydroperoxide accumulation in xCT^KO^ PDAC cells co-cultured with other PDAC wt cells or in media supplemented with an alternative cysteine-donor—N-acetylcysteine (NAC). (**B**,**C**). Phenotype of MiaPaCa-2 xCT^KO^ cells co-cultured with different xCT-expressing cells (LS174T, A549 and Capan wt) in media +/− the low dose of pro-oxidant, glucose oxidase (0.2 mU/mL GOx): B. BODIPY 591/581 C11 staining; (**C**). Cell death. (**D**). MiaPaCa-2 wt and xCT^KO^ cells were cultivated for 24 h in DMEM +/− 1 mM NAC, or in conditioned media (CM) of LS174T/A549 wt. Changes in phosphorylation status and protein abundance of members of the two major AA-sensing pathways GCN2 (p-GCN2/ATF4) and mTORC1 (p-S6K1 and p-RPS6) were analyzed by Western blot. All experiments have been performed in triplicate and the representative blots and histograms are shown. Bar graph shows mean ± SEM; n = 3; *, *p* < 0.05, comparison with WT control group; ^#^, *p* < 0.05, comparison with the corresponding untreated group.

Considering the rescue effect observed with HDF cells, we then determined if this result is fibroblast-specific or rather a universal phenomenon among all wt (xCT-expressing) cells. Co-culture of MiaPaCa-2 xCT^KO^ with Capan-2 wt cells proved to have the same rescue effect as observed for co-culture with HDFs (Figure 2A). Similar results were obtained when Capan-2 xCT^KO^ cells (described in [10]) were cultured with MiaPaCa-2 wt counterparts (Figure 2A). Similarly, co-culturing MiaPaCa-2 xCT^KO^ cells (clones #1 and #2) with wt cells of different tumor origin (lung carcinoma—A549, colon adenocarcinoma—LS174T) had exactly the same effect (Figure 2B). However, this effect was significantly diminished in a more oxidative environment that has been achieved by addition of the oxidizing agent-glucose oxidase (GOx). GOx is an enzyme that catalyzes the oxidation of β-D-glucose using molecular oxygen (O_2_) to gluconic acid, yielding hydrogen peroxide (H_2_O_2_) as a by-product [19]. The results of BODIPY 591/581 C11 staining and FACS analysis showed that in the presence of GOx, the saving effect of the host cell lines was significantly reduced, leading to accumulation of the lipid hydroperoxides in xCT^KO^ cells (Figure 2B). The cell death after 48 h of co-culture was observed under the optical microscope and measured by PI staining and FACS analysis (Supplementary Figure S1A, Figure 2C). The results agreed with the lipid hydroperoxides staining, showing increased cell death of the MiaPaCa-2 xCT^KO^ in the presence of GOx, even if A549, LS174T or Capan-2 wt host was present, suggesting that the “saving agent” exported by the xCT-expressing cells is susceptible to oxidation (Figure 2B,C). It is important to point out that GOx had no effect on either guest or host wt cells (Figure 2B,C).

As the genetic ablation of xCT induces cellular cysteine depletion and triggers an AA-stress response in PDAC cells [10], the status of the two main pathways of AA sensing, GCN2-ATF4, and mTORC1 were examined by Western blot after 24 h of cultivating MiaPaCa-2 xCT^KO^ (clones #1 and #2) in CM from LS174T or A549 cells. The results presented in Figure 2D corroborate previously published observations, exhibiting that the ablation of cystine uptake by disruption of xCT transport promotes high activation of GCN2-ATF4 pathway, as shown by the increased level of p-GCN2 and ATF4 in the CTL group of xCT^KO^ in comparison with wt and decreased mTORC1 signaling, as observed by the decrease of phosphorylation of S6 kinase 1 (S6K1) and ribosomal S6 protein (S6RP), two well established targets of the mTORC1 signaling pathway. These effects were completely reversed in the presence of NAC, but also when the cells were cultivated in the CMs of A549 and LS174T. Besides, co-culture of MiaPaCa-2 xCT^KO^ cells either with LS174T or A549 wt cells restituted redox balance to the same extent as NAC, seen through measurement of total ROS level (Supplementary Figure S1B)

Interestingly, Figure 2D also shows higher expression of CHAC1 (glutathione-specific γ-glutamylcyclotransferase 1), a cytoplasmic enzyme that catalyzes the cleavage of GSH into 5-oxo-L-proline and Cys-Gly dipeptide, in the xCT^KO^ cells in control conditions, likely due to an effort to liberate cysteine from its main storage—GSH. Similar to AA-sensing pathway, this effect was also reversed with CM of wt cells or in the presence of NAC.

### 2.3. Cystine-Cysteine Shuttle Fuels Cooperation between wt and xCT^KO^ Cells

**Figure 3:**
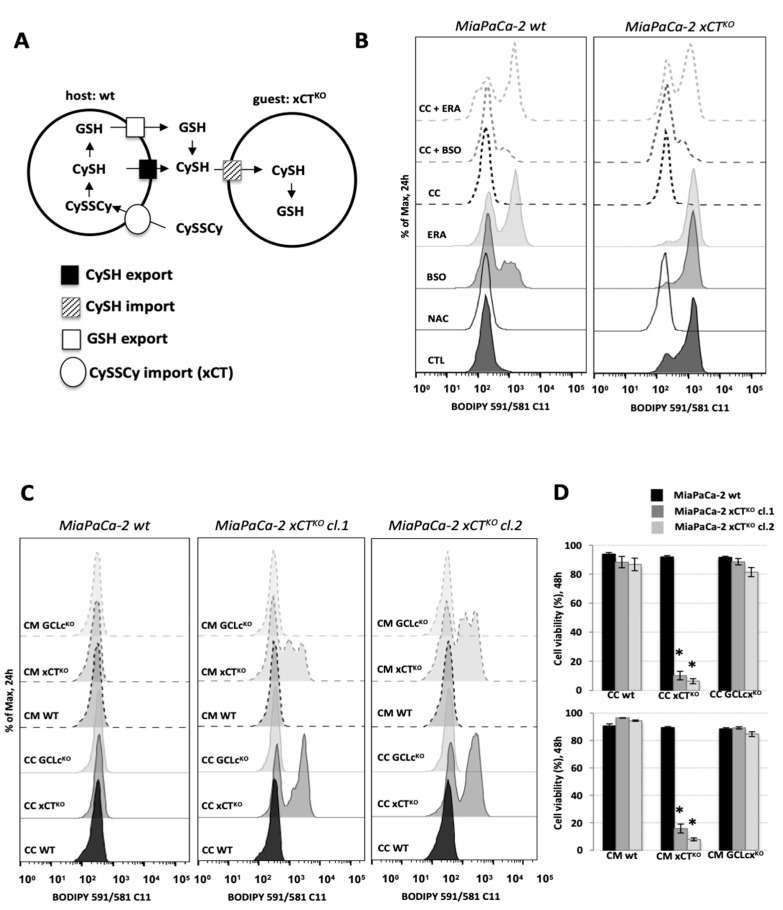
Cystine-cysteine shuttles fuel cooperation between WT and xCT^KO^ cells. (**A**). Schematic representation of the cystine-cysteine (CySSCy, CySH) shuttle: xCT-expressing cells (“host”) are able to import the oxidized form of cysteine, reduce it and export it. Or, in the alternative scenario, the “host” cells can synthesize and export glutathione (GSH), which then will be cleaved outside the cell to the constituent amino acids. Cysteine provided in one or both ways is taken up by the xCT^KO^ cells (“guests”) maintaining the amino acid and redox balance. (**B**). MiaPaCa-2 wt or xCT^KO^ cells alone (full outer lines of the histograms) or in the co-culture with A549 wt (CC—dashed outer lines of the histograms) counterparts in DMEM media +/− 1 mM N-acetylcysteine (NAC), 100 μM inhibitor of GSH biosynthesis (buthionine sulphoximine, BSO) or 1 μM inhibitor of xCT, erastin. (**C**)**.** Accumulation of lipid hydroperoxides and (**D**). Cell viability of MiaPaCa-2 xCT^KO^ cells in co-culture (CC) or cultivated in the presence of conditional media (CM) of Capan-2 GCLc^KO^ or xCT^KO^ cells during (**C**) 24 h and (**D**) 48 h. All experiments have been performed in triplicate and the representative histograms are shown. Bar graph shows mean ± SEM; n = 3; *, *p* < 0.05, comparison with WT control group.

Figure 3A proposes a hypothetical model of how the interplay between xCT-expressing cells and xCT^KO^ cells could be functioning. As host cells express xCT transporters, they are able to import cystine (CySSCy), abundantly available in normal DMEM medium, and once inside the host cells, cystine is reduced to cysteine (CySH). Following this, CySH can be exported by the host cells into the extracellular space and then taken up by the guest cells via different transporters. In an alternative and more complex scenario, reduced CySH can be incorporated into GSH by the host cells, then exported and cleaved extracellularly. The Cys-Gly dipeptide produced by the cleavage of GSH can be taken up by the guest cells via different transporters, such as the low-capacity/high-affinity proton-coupled cotransporter of diverse di- and tri-peptides transporter (PEPT2, not shown in the scheme), or further broken down into individual AA by the action of dipeptidases and imported into xCT^KO^ cells by cysteine transporters. The latter mechanism is known in the literature under the name “Meister cycle” or γ-glutamyl cycle.

In order to understand the role of extracellular cystine and intracellularly produced GSH in this rescue phenomenon, the ferroptotic phenotype of the KO cells co-cultured with A549 wt cells was assessed in the presence of buthionine sulphoximine (BSO), an inhibitor of GSH biosynthesis [20], or erastin (ERA). Treatment of MiaPaCa-2 wt cells with 100 μM BSO or 1 μM ERA promoted high accumulation of lipid hydroperoxides in these cells (Figure 3B), while their effects on the A549 wt cells was negligible (Supplementary Figure S2A). Furthermore, the saving effect of the co-culture (dashed lines) was partially disturbed in the presence of BSO and completely prevented by the addition of ERA. These results suggests that (1) there is a key role of cystine import via xCT and (2) there is a dispensable role of GSH in the phenomenon (Figure 3B).

To further investigate this hypothesis, the knockout of the catalytic subunit of GCL enzyme (Capan-2 GCLc^KO^), as well as the Capan- 2 xCT^KO^ (both previously described, [10]) were used as host cells in a co-culture assay with MiaPaCa-2 xCT^KO^. Both the accumulation of membrane lipid hydroperoxides after 24 h (Figure 3C) and cell death after 48 h (Figure 3D) were measured. We observed that an inability of the host cells to synthetize GSH (GCLc^KO^) had no impact on supporting xCT^KO^ cell survival, as represented by the lack of lipid hydroperoxides accumulation, both in the condition of co-culture (full lines) or when MiaPaCa KOs were cultivated in conditional medium (CM—dashed lines). Additionally, no cell death after 48 h was detected in this condition (Figure 3D). On the other hand, MiaPaCa-2 xCT^KO^ cells co-cultured with Capan-2 xCT^KO^ or cultivated in CM of these cells exhibited an increase in the membrane lipid hydroperoxide accumulation (Figure 3C—full and dash lines, respectively) and a high rate of cell death, about 90%, after 48 h (Figure 3D). These observations indicate the importance of xCT expression, and the consequently CySSCy import, by host cells in order to support the survival of the guest cells. Indeed, when MiaPaCa-2 xCT^KO^ cells were co-cultured with A549 wt in media containing no cysteine/cystine, similar results were obtained; the absence of CySH/CySSCy disrupted the rescuing effect of the host cells towards the guest cell, as showed by the accumulation of lipid hydroperoxides on the membrane of xCT^KO^ cells after 24 h (Supplementary Figure S2B). It is important to point out that the depletion of cysteine did not affect the host cells (A549 wt) during 24 h in this regard (Supplementary Figure S2B). Additionally, worth noticing here is that both Capan xCT^KO^ and GCLc^KO^ cells were viable during the 3 days period experimentation. The effects of the conditional media as well as effects of the host cell proliferation on the MiaPaCa-2 xCT^KO^ cells rescue have been validated in the experiments with the Boyden chamber (Supplementary Figure S3).

### 2.4. Genetic Disruption of the ASCT2 Transporter Affects Cooperation between xCT-Expressing and xCT^KO^ Cells

**Figure 4:**
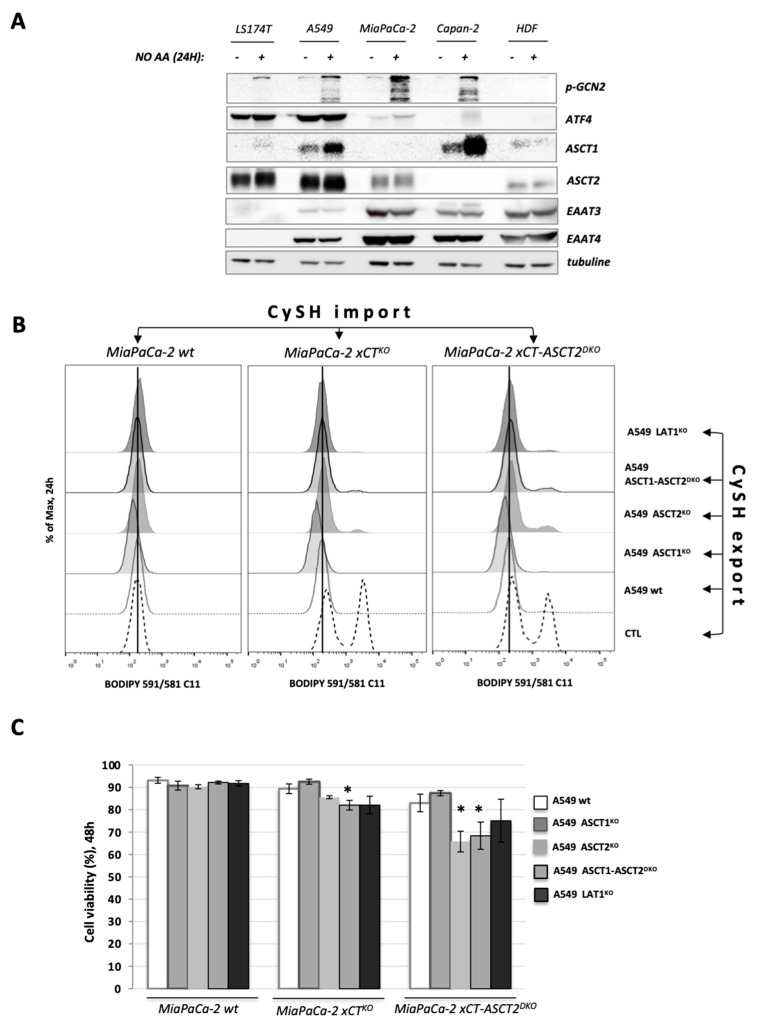
Genetic disruption of the ASCT2 cysteine transporter affects cooperation between xCT-expressing and xCT^KO^ cells. (**A**). A variety of xCT wt cells (LS174T, A549, MiaPaCa-2, Capan-2 and HDF) were grown in DMEM media +/− amino acids during 24 h and protein content of different cysteine transporters (ASCT1 (SLC1A4), ASCT2 (SLC1A5), EAAT3 (SLC1A1), EAAT4 (SLC1A6) were analyzed by Western blotting. (**B**,**C**). Lipid hydroperoxide accumulation and cell viability of MiaPaCa-2 xCT^KO^ and xCT-ASCT2^DKO^ cells (guest cells—CySH import) in control conditions or co-cultured with 6% A549 wt, ASCT1^KO^, ASCT2^KO^, ASCT1-ASCT2^DKO^, or LAT1^KO^ (host cells—CySH export). All experiments have been performed in triplicate and the representative blots and histograms are shown. Bar graph shows mean ± SEM; n = 3; *, *p* < 0.05, comparison with WT control group.

Substrate promiscuity of amino acid transporters allows cysteine to be transported through the cell by a variety of neutral amino acids carriers such as alanine-serine-cysteine (ASCTs), sodium-coupled neutral amino acid (SNATs) or the excitatory amino-acid (EAATs) transporters, well known Na^+^-dependent amino acid exchangers/importers [21]. The expression levels of these probable cysteine transporters on 4 cancer cell lines (LS174T, A549, MiaPaCa-2 and Capan-2), and their regulation in an AA-starvation condition were verified by Western blot. ASCT1 (SLC1A4) and ASCT2 (SLC1A5) transporters exhibited high expression in most cancer cell lines, with the exceptions of ASCT1 and ASCT2 that were virtually undetectable in MiaPaCa-2 or Capan-2, respectively (Figure 4A). Concerning members of the EAAT transporter family, detection of EAAT3 and EAAT4 was not observed in LS174T cells. Additionally, the expression of EAAT3 in A549 cell line was lower in comparison with other cell lines. Additionally, no up-regulation of EAAT carriers was observed in AA-stress condition. Curiously, the transcriptional factor ATF4 (activating transcription factor 4) seems to be overexpressed in LS174T and A549 cells independently of AA-starvation and GCN2 activation when compared to the other cell lines, possibly due to an eiF2a independent induction of ATF4 such as mTORC1 [22] (Figure 4A).

Considering the high expression of the members of ASCT family in the cell lines used in this work and in order to understand the involvement of these carriers on the uptake of CySH by the guest cells to sustain their survival, a knockout of ASCT2 was obtained in MiaPaCa-2 xCT^KO^, hereafter referred to as MiaPaCa-2 xCT ASCT2^DKO^ (Supplementary Figure S4A). Moreover, since no expression of ASCT1 was observed in MiaPaCa-2 cell line (Figure 4A), even under AA-starvation, this mutant is considered a triple knockout of xCT-ASCT1-ASCT2.

Regarding the host cells, CRISPR-Cas9 was again used to obtained single knockouts of ASCT1 and ASCT2 (previously described in [23]) or the double knockout of ASCT1-ASCT2 in A549 cells. The total ablation of the expression of targeted proteins was validated via Western blot (Supplementary Figure S4B) and two clones of each KO were used on subsequent experiments to minimize clonal effects. Additionally, as Simmons-Willis et al. [24] demonstrated that methylmercury (MeHg) is transported by the L-type AA transporter-1 (LAT1/SLC7A5) when bound to CySH in a complex (MeHG-L-cysteine), the A549 LAT1^KO^, previously described by Cormerais et al. [23] was also used to verify if LAT1 carriers could be involved in the host–guest interplay.

The impact of ASCT1 and/or ASCT2 as well as LAT1-disruption on A549 cells was measured by the ability of these cells to form clones by a clonogenicity assay (Supplementary Figure S4C). Under control conditions (DMEM (CySSCy) + FBS) A549 ASCT2^KO^ exhibited a decrease in the number of clones, compared to A549 wt or ASCT1^KO^. ASCT1^KO^ cells showed no difference in the clonal capacity compared to wt, possibly due to a higher adaptive expression of ASCT2 in ASCT1^KO^ cells, as observed by a slight increase of the ASCT2 band on the Western blot (Supplementary Figure S4B). Moreover, the disruption of both ASCT1-ASCT2 transporters significantly inhibited clonal formation of A549 cells, demonstrating that the single inhibition of ASCT1 or ASCT2 leads to an adaptive response to overcome the disturbance on the AA pool necessary to sustain cell proliferation. This proliferative inhibition was also observed in LAT1^KO^, as previously observed by Cormerais et al. [23].

We then investigated if ablation of the above-mentioned transporters can affect the export from the host and the import into the guest cells of the “saving” agent, i.e., cysteine. All experiments were performed with two independent clones of each KO or DKO, to minimize clonal heterogeneity. However, for simplicity only one KO is presented in the main figures, while the rest of the results can be found in the Additional Supplementary data file. As host cells, A549-derived cell lines were pre-seeded and after 24 h, co-cultured with MiaPaCa-2 wt, xCT^KO^, or xCT-ASCT2^DKO^ (guest cells). Following 24 h, the membrane lipid hydroperoxide accumulation was measured (Figure 4B). We observed an expected increase in lipid hydroperoxides accumulation in the CTL condition (absence of host cells) of MiaPaCa-2 xCT^KO^ and xCT-ASCT2^DKO^ cells and a complete reversal of this phenotype when A549 wt are present (Figure 4B). Interestingly, the co-culture of MiaPaCa-2 xCT^KO^ or xCT-ASCT2^DKO^ with A549 ASCT2^KO^ or ASCT1-ASCT2^DKO^ did not completely rescue the accumulation of membrane lipid hydroperoxides, while A549 ASCT1^KO^ was as effective as A549 wt, suggesting the importance of the host ASCT2 transport activity for the export of the CySH to the culture medium. Regarding the guest cells, the knockout of ASCT2, concomitantly with very low or no expression of ASCT1, seems to have an impact on the import of this CySH when in the co-culture with A549 ASCT2^KO^ or ASCT1-ASCT2^DKO^, as demonstrated by a marginally higher accumulation of lipid hydroperoxides (Figure 4B). Table 1 summarizes different combinations of the mutations of the “host” and “guest” cells and their effect on the lipid peroxidation and cell death.

Further, in order to investigate the importance of these transporters for the basal import (independently of co-culture context) of CySH by A549 cells (host), clonogenicity assays were performed in DMEM medium supplemented exclusively with cystine (CySSCy, 0.2 mM) or reduced cysteine (CySH, 0.4 mM) plus dialyzed FBS (dFBS, in order to prevent any type of AA “contamination” coming from the serum). The specific inhibitor of SNAT transporters family, 2-(methylamino)isobutyric acid (MeAIB), was used to potentialize the requirement of ASCT activity for import of CySH. However, no differences were found compared to the control conditions (CySSCy/FBS), suggesting that individual disruption of ASCT1 or ASCT2 does not influence the CySH import by A549 cells (Supplementary Figure S4C). Unfortunately, no clones were observed in A549 ASCT1-ASCT2^DKO^ in either CySSCy or CySH conditions when dialyzed serum was used, thus it was impossible to conclude if and to what extent the deletion of both transporters impairs CySH uptake by these cells.

Despite exhibiting an accumulation of lipid hydroperoxides (Figure 4B), this increase seems to be insufficient to drive MiaPaCa-2 xCT^KO^ cells co-cultured during 48 h with A549 ASCT2^KO^ cells to ferroptotic cell death, as demonstrated by cell viability assays (Figure 4C, Supplementary Figure S5). Although minor, but significant ferroptotic cell death was detected in MiaPaCa-2 xCT^KO^ co-cultured with A549 ASCT1-ASCT2^DKO^. Corroborating with the BODIPY staining, MiaPaCa-2 xCT-ASCT2^DKO^ co-cultured with A549 ASCT2^KO^ and ASCT1-ASCT2^DKO^ exhibited significant cell death after 48 h (35 and 32%, respectively). Altogether, these results indicate the importance of ASCT2 carriers to both, the export of CySH by the host cells to the external culture medium and to the import of this CySH by the guest cells. The capacity of xCT-expressing cells to support xCT^KO^ cells seems to be affected by an accumulative impairment when ASCT2 carriers are not expressed by host and guest cells.

As far as LAT1^KO^ of the host cells is concerned, the effects were visible only at the level of MiaPaCa-2^DKO^ at the level of lipid hydroperoxides, with no effect on the survival of the guest cells, suggesting that LAT1 is dispensable in this regard.

### 2.5. Different Cysteine Transporters Are Necessary for “Guest–Host” Intercommunication

CRISPR-Cas9 technology is a powerful approach to generate genetic models for fundamental research, shedding light on the understanding of structure, function and regulation of specific proteins. However, it is important to bear in mind that some biological considerations such as the adaptive response of the cell undergoing these genetic modifications. In order to overcome this issue and validate the results obtained previously, a pharmacological approach was also addressed.

**Figure 5:**
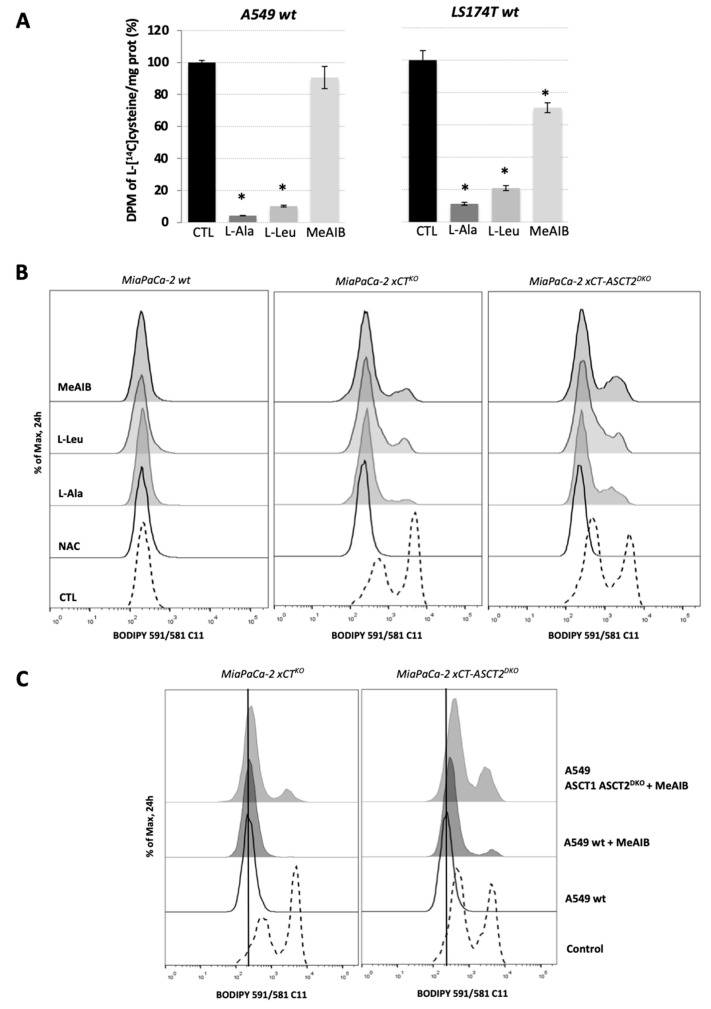
Multiple cysteine transporters are necessary for “guest–host” intercommunication. (**A**). Cysteine transport activity of A549 wt and LS174T wt cells was measured by the uptake of [^14^C]- 1, 2, 1′, 2′-cystine (^14^C-cystine) reduced by 100 μM β-mercaptoethanol in HBSS media containing 50 μM cold cystine and supplemented or not with 10 mM L-alanine, L-leucine or MeAIB. These results represent the average ± SEM, n = 2, comparison with WT control cells, * *p* < 0.05. (**B**). Accumulation of the lipid hydroperoxides in MiaPaCa-2 wt, xCT^KO^ or xCT-ASCT2^DKO^ (guest cells) co-cultured with A549 wt cells (host cells) in the presence or not of the 1 mM NAC, 3 mM L-alanine, 3 mM L-leucine or 3 mM MeAIB after 24 h. (**C**)**.** Lipid hydroperoxides levels measured by BODIPY 591/581 C11 staining and FACS analysis of MiaPaCa-2 xCT^KO^ and MiaPaCa-2 xCT-ASCT2^DKO^ (guest cells) co-cultured for 24 h with A549 wt or A549 ASCT1-ASCT2^DKO^ (host cells) in the presence or not of SNAT inhibitor, MeAIB; Control condition represents the phenotype of guest cells in the absence of host cells. Representative histograms of three independent experiments are shown.

The overall cysteine transporter capacity was measured by the intracellular accumulation of L-[1,2,1′,2′-^14^C]cysteine (^14^C-cystine) reduced by 100 μM β-mercaptoethanol in A549 and LS174T wt cells in the presence of 10 mM of competitive inhibitors of the ASCT family, L-alanine (L-Ala); LAT1, L-Leucine (L-Leu); or SNAT family, MeAIB. The results showed that when applied acutely, L-Ala inhibits about 90-95% whereas L-Leu suppresses the ^14^C-cysteine import for about 80–90% in A549 and LS174T cells (Figure 5A). The presence of 10 mM MeAIB decreased the import of ^14^C-cysteine by 25%, approximately, in LS174T cells but did not promote any significant effect on A549 wt cells (Figure 5A). These results suggest that in its reduced form, cysteine (CySH), is imported by the cells mainly through the LAT1 carrier and ASCT family of transporters.

In order to determine if the CySH exchange between host and guest cells can be impaired by acute, pharmacological inhibition of ASCT, LAT, and SNAT families of transporters, the ferroptotic phenotype of MiaPaCa-2 xCT^KO^ and MiaPaCa-2 xCT-ASCT^DKO^ was measured by the accumulation of lipid hydroperoxides after 24 h of co-culture with A549 wt cells in the presence of 3 mM of the inhibitors: L-Ala, L-Leu, or MeAIB. Corroborating with the results in Figure 4B, a slight inhibition of the cooperation between MiaPaCa-2 xCT^KO^/xCT-ASCT2^DKO^ and A549 wt cells (Figure 5B) were impaired in the presence of ASCT inhibitor L-Ala. Similar results were obtained by the co-culture of guest cells with wt host cells in the presence of L-Leu and MeAIB. The inhibition of LAT1, by high concentrations of leucine, or SNAT, by MeAIB, promoted lipid hydroperoxide accumulation on xCT^KO^ or ASCT2-xCT^DKO^ cells as shown by the shift and increase of the peaks related to BODIPY C11 staining (Figure 5B). The most surprising result was that almost none of these conditions had significant effect on the survival of guest cells (Supplementary Figure S6), probably due to time-dependent adaptation of the cells to the inhibition of individual transporters. Altogether, the results suggest that the CySH shuttle is not carried by a single transporter, but rather is a result of a synergistic activity involving highly expressed AA-transporters in the cells.

In fact, this synergistic activity was best visible when MiaPaCa-2 xCT^KO^ or xCT-ASCT2^DKO^ was co-cultured with A549 ASCT1-ASCT2^DKO^. Compared to the co-culture with A549 wt, ASCT1-ASCT2-disrupted host cells had lower rescuing effects, as shown by increase in lipid hydroperoxides (Figure 4B). Additionally, MeAIB potentiated the accumulation of lipid hydroperoxides in the co-culture of host cells and double knockout guest cells, showing the importance of SNAT transporters on the import of CySH by these cells when ASCT2 is not functional [25] (Figure 5C).

## 3. Discussion

In our previous study we showed that PDAC cell lines (MiaPaCa-2 and Capan-2), in in vitro conditions, are extremely sensitive to the genetic or pharmacological inhibition of the cystine transporter, xCT [10]. This was in complete accordance with other studies showing xCT as a main player in ferroptotic cell death in a variety of cancer types (reviewed in [26]). However, the results obtained with the mouse xenograft model surprisingly contradicted those obtained in in vitro conditions. Namely, although xCT^KO^ cells were not able to survive in in vitro conditions, unless an alternative donor of cysteine (such as NAC) was added, in vivo they not only survived, but after a slight delay, developed tumors growing at the same rate as their WT counterparts [10]. Even more, when these tumor cells were returned to culture conditions, they exhibited the exact same phenotype as before the injection into the mice: NAC-dependency, accumulation of lipid hydroperoxides, and ferroptosis [10], suggesting that no novel stable adaptation (e.g., mutation) had been acquired.

The reliance of the cancer cells on an extracellular source of cysteine has been demonstrated in the elegant work of Cramer et al. [27] who used the enzyme cyst(e)inase to catalyze the systemic depletion of the plasma cyst(e)ine pool in mice and non-human primates. A series of studies showed that enzymatic degradation of extracellular cyst(e)ine selectively induces cell cycle arrest and ferroptosis in cancer cells [15,27,28,29]. Hence, in vivo survival and proliferation of xCT^KO^ cells, which are unable to import the oxidized form of cysteine, could be explained only by the use of the reduced form of this amino acid. One of the possible sources of the reduced cysteine is the plasma. However, quite surprisingly, supplementation of NAC in the drinking water (in the concentration that has been proven as potent in the case of c-Myc-driven tumors in vivo [30]) had no effect on the tumor growth kinetic of the xCT^KO^ cells in vivo, suggesting that circulation might not be the main source of cysteine in this context [10]. Cysteine could also be provided from the neighboring fibroblasts, as it has been already shown for chronic lymphocytic leukemia or ovarian cancer cells expressing low levels of xCT [31,32]. Besides, the importance of the fibroblast feeder layer for cancer cell survival has been phenomenon known for a long period of time [33]. Interestingly, in the tumor masses isolated from mice, we detected stromal fibroblasts in addition to the initially injected xCT^KO^ cells [10] which led us to hypothesize that the main source of cysteine for xCT^KO^ cells indeed might be neighboring stromal fibroblasts. To investigate this issue, we used co-culture with the xCT-expressing HDF cells, which themselves proved to be highly sensitive to ferroptosis induced by xCT-inhibition (Figure 1A).

Co-culturing of MiaPaCa-2 xCT^KO^ cells with 15 times less HDF cells completely reversed the characteristic phenotype of the former observed through the lipid hydroperoxide accumulation and survival after 24 h and 48 h, respectively (Figure 1B). These results suggested that presence of HDFs could ‘de-sensitize’ MiaPaCa-2 to ferroptosis. However, in recent years an accumulating number of studies have suggested that increased cell density increases the resistance of cancer cells to this type of cell death through cell-to-cell contact and consequent activation of the Hippo pathway (for further reading see [34]). In order to exclude this possibility, only CM of the HDF was used, and the same rescue effect of the MiaPaCa-2 xCT^KO^ cells had been observed. These data unequivocally showed that the “saving agent” is a small (less than 3 kDa), redox-sensitive molecule; as well as that the “saving phenomenon” is not fibroblast-specific but a general phenomenon of many different xCT-proficient cells (such as colon or lung adenocarcinoma) Figure 1B,C and Figure 2).

In the report of Wang et al. [32] cooperation between ovarian cancer cells and fibroblasts upon cisplatin treatment has been ascribed: 1) to xCT presence in the plasma membrane of fibroblasts which allows them to import as much cystine as possible from the extracellular space, and 2) to both cysteine and GSH provided from cystine metabolism in the fibroblasts (Figure 3A). According to our Western blot analysis of the main components of the AA-sensing pathways, CM of the “host” cells restored AA balance within the xCT^KO^ cells to the same extent as NAC, further proving restoration of the cysteine pool in these cells by the “rescue agent” (Figure 2D). However, cysteine could be provided from GSH degradation, which is reflected by strong induction of CHAC1 in xCT^KO^ in the control conditions. CHAC1 is an intracellular enzyme responsible for the GSH breakdown, and thus, release of the cysteine from its main storage molecule [35]. Interestingly, the level of CHAC1 was returned to the control when the xCT^KO^ cells were cultured in the host’s CM, suggesting a different mechanism of cysteine supply. This, however, still does not prove that GSH synthesized in the fibroblast is not important for the phenomenon, as it could be exported and degraded extracellularly giving rise to extracellular cysteine (reviewed in [36]). In order to address this issue, as well as dispensability of the host‘s xCT in our system, we investigate the influence of erastin (inhibitor of xCT) and BSO (inhibitor of GSH synthesis) on the wt-to-xCT^KO^ cooperation as well as the “rescue” potential of xCT^KO^ or GCLc^KO^ as “host” cells. Data obtained with the pharmacological approach unequivocally showed that host’s xCT plays a fundamental role in the phenomenon, while BSO did not prevent interplay to the same extent (Figure 3B). Similar results were obtained with the genetic approach, whether co-culture with or CM of xCT/GCLc^KO^ was used (Figure 3C, D). Our data are in line with what was obtained previously when Mandal et al. showed that GSH depletion in mammalian cells can be easily overcome by forced expression of xCT and consequent increase in cysteine intracellular content, which allows maintenance of the redox balance within the cells [37].

From this first part of in vitro study, we can conclude that, although oxidized cysteine is the major/dominant form of the amino acid in the circulation and extracellular milieu, cystine-cysteine cycle is a powerful mechanism exploited by low- or non- expressing xCT cells to prevent ferroptosis. However, this does not diminish the importance of xCT in the context of ferroptosis prevention; but on the contrary, it once again emphasizes xCT-mediated cystine import as a fundamental step in the cycle and thus in the ferroptosis axis. These results are in great accordance with the manuscript of Banjac et al. [38] published before the term “ferroptosis” was coined, in which the expression level of xCT were shown to determine the chemoresistance of Burkitt’s lymphoma by fueling a highly efficient cystine/cysteine redox cycle. The different question that has been imposed at this point is how reduced cysteine is exported from the host cells and imported into the guest cells and if it is possible to block the cysteine-cystine cycle by interfering with these cooperating transporter systems. Literature data regarding the transport of reduced cysteine pale greatly in comparison with those dealing with the transport system for its oxidized counterpart—cystine. The alanine-serine-cysteine (ASCT) family of transporters has been reported to be the most important in this context [39,40]. Hence, we decided to investigate the significance of both ASCT1 and ASCT2 transporters in the cysteine shuttling between host and guest cells by using a genetic approach (Figure 4A). Quite surprisingly, ablation of these transporters either in host or guest cells or in both had almost no effect on their cooperation (Figure 4B,C, Supplementary Figure S5). Modest effects were observed in the case where ASCT2 was lacking from the guest cell population, suggesting that it could be, to a certain degree, involved in the import part of the shuttle (Figure 4B, C).

When it comes to investigating amino acid transporters, a genetic approach, although it provides valuable information regarding their role and dispensability, still has a major limitation reflected in the adaptive strategies developed by the cells in response to the long-lasting selective pressure. This arose as a concern considering that none of the KOs/DKOs showed any phenotype when grown in the media containing exclusively reduced or oxidized cysteine (Supplementary Figure S4C). Potential explanation for this might lie in a wide range of transporters capable of transporting cysteine across membrane, which could fully take over the role of cysteine transport, especially in the cells with genetically disrupted ASCTs. Namely, studies suggest that except ASCT family of transporters, sodium-coupled neutral amino acid (SNAT) and L-amino acid (LAT) transporter families could be involved in the cysteine transport [24,41]. To address this issue, instead of ASCT-genetically modified cells we used competitive inhibitors of the ASCT family (L-Ala), but also LAT1 and SNAT family of transporters (L-Leu and MeAIB, respectively). Both L-Ala and L-Leu had profound suppressive effects on the cysteine transport across the membrane (Figure 5A). This finding appears to be the first demonstration that LAT1, the transporter for essential amino acids is also capable to directly transport cysteine as previously suggested [40]. However, when co-culture of MiaPaCa-2 xCT^KO^ or xCT-ASCT2^DKO^ and A549 wt was maintained in the presence of these inhibitors, very similar effects were observed as in the case of genetic approach (Figure 5B). Collectively these results unequivocally demonstrated that acute inhibition of cysteine transport is achievable, but at the same time, it is also easily overcome due to a wide range of transporters capable of transporting this amino acid. This further suggests that the selectivity for reduced cysteine is dispersed among different transporters, and consequently, one transporter-targeted-strategy seems inapplicable. However, our data also point out that targeting ASCT and SNAT families together might disrupt cysteine transport, as the cysteine/cystine shuttle was significantly affected if the co-culture of xCT-ASCT2^DKO^ and ASCT1-ASCT2^DKO^ was maintained in the presence of SNAT inhibitor (Figure 5C).

## 4. Conclusions

Collectively, the results of the present study clearly show that a cystine-cysteine cycle provides maximal exploitation of the extracellular cyst(e)ine pool, which allows maintenance of redox and amino acid balance and prevention of ferroptosis within the tumor cells. Although, this mechanism seems to be used by the xCT^KO^ cells to survive and thrive in vivo, our data unequivocally demonstrate that the presence of any cells expressing xCT within the tumor mass is condition sine qua non for the cycle to run smoothly. On the other side, the role of GSH as a potential donor of reduced cysteine seems to be dispensable in our system; however, we cannot exclude the possibility that a role for GSH would be important in some other contexts, such as for cancer cells with low or no GSH biosynthesis. Additionally, to the best of our knowledge, this is the first study that examined the role of individual transporters in cysteine transport in cancer cells. According to the data, cysteine appears to be imported through several transporter systems (ASCT1/2, SNATs, LAT1), perhaps reflecting the essentiality of this unique amino acid engaged in: proteogenic, protein folding, oxidative stress and ferroptosis ‘repression’.

## 5. Materials and Methods

### 5.1. Cell Culture

Human dermal fibroblast (HDF) and lung carcinoma A549 cells were obtained from ATCC (Manassas, VA, USA); MiaPaCa-2 and Capan-2 cells were kindly provided by Dr. Sophie Vasseur (CRCM, Marseille, France) and LS174T colon adenocarcinoma cells were kindly provided by Dr. Van de Wetering. MiaPaCa-2 and Capan-2 xCT-disrupted cells and Capan-2 GCLc^KO^ were obtained and described by Daher et al. [10]. The knockouts of ASCT2 and LAT1 transporters in A549 cells were obtained and described by Cormerais et al. [23]. The cells have been authenticated and routinely tested for Mycoplasm (PlasmoTest Mycoplasma Detection Kit; InvivoGen). Cells were cultivated at 37 °C and 5% CO_2_ in DMEM (Gibco, Gaithersburg, MD, USA) supplemented with 7.5% FBS, penicillin (10 U/mL) and streptomycin (10 μg/mL). The stock cells of xCT^KO^, ASCT1^KO^, ASCT2^KO^, ASCT1-ASCT2^DKO^, or xCT-ASCT2^DKO^ cells were cultivated in the presence of 1 mM N-acetylcysteine (NAC; A9165; Sigma-Aldrich, Munich, Germany).

### 5.2. Genetic Ablation of Potential Cysteine Transporters

Human A549 wt, ASCT2^KO^ or MiaPaCa-2 xCT^KO^ cells were transfected, via Nucleofection (Lonza), with pSpCas9(BB)-2A-GFP (PX458) plasmids (kindly obtained from Dr. Feng Zhang, Addgene plasmid #48138) containing CRISPR-Cas9 with small guide RNA (sgRNA) targeting the exons 1 and 6 of SLC1A4 (ASCT1). The gRNAs were designed using the CRISPR Design Tool (http://crispr.mit.edu, assessed on 5 July 2019). The GFP positive cells were sorted by single-sorting FACS analysis (BD FACSMelody™, Allschwil, Switzerland), after 24 h post-transfection, and seeded in 96-well plate containing DMEM supplemented with 7.5% FBS and 1 mM NAC. Screening of the clones via Western blot assessed the validation of the knockouts and two different KO clones for each cell line were chosen for subsequent experiments, to minimize the clonal heterogeneity. However, since the findings obtained were similar for the two clones, we showed the results of one of the clones for both cell lines for simplicity reasons.

### 5.3. Clonogenicity Assay

A549 wt, ASCT1^KO^, ASCT2^KO^, ASCT1-ASCT2^KO^, and LAT1^KO^ were seeded in low density (1.000 cells) in 60 mm dishes and incubated for 15 days at 37 °C/5% CO_2_ with normal DMEM media (Gibco) supplemented with 7.5% FBS (control condition) or depleted DMEM (high glucose, no glutamine, no methionine, no cysteine or cystine; 21013-024; Gibco) supplemented with 1× GlutaMax (Gibco), 100 mM L-methionine (M9625, Sigma-Aldrich, Munich, Germany), 100 mM sodium pyruvate (Gibco), 10% dialyzed FBS in the presence or not of L-cysteine (CySH; C7352; Sigma Aldrich) or L-cystine (CySSCy; C8755; Sigma Aldrich). Following, the cells were stained with 5% Giemsa (Fluka, Buchs, Switzerland) for 30 min. For this experiment, FBS was dialyzed against PBS 1× for 24 h under constantly agitation.

### 5.4. Co-Culture and Conditional Media

Regarding the co-culture, two terminologies were adopted in this work in order to simplify the explanation of the results. HDF wt, MiaPaCa-2 wt, Capan-2 wt, Capan-2 GLCc^KO^, Capan-2 xCT^KO^, LS174T wt, A549 wt, A549 ASCT1^KO^, A549 ASCT2^KO^, A549 ASCT1-ASCT2^KO^ or LAT1^KO^ were called “host cells” and pre-seeded at low density (10 000 cells/well) in 6-well dishes and cultivated in DMEM medium supplemented with 7.5% FBS at 37 °C/5% CO_2_ during 24 h. Following, the “guest cells” (MiaPaCa-2 wt, xCT^KO^ or xCT-ASCT2^KO^) were seeded in the same wells at high density (150 000 cells) for 24 h or 48 h. When indicated, the cells were incubated in the presence of glucose oxidase (0.2 mU/mL GOx; G6125; Sigma Aldrich), buthionine sulphoximine (100 μM; BSO; Sigma Aldrich), erastin (1 μM; E7781; Sigma Aldrich), L-alanine (3 mM; A7627; Sigma Aldrich), L-leucine (3 mM; L8000; Sigma Aldrich) or N-methylaminoisobutyric acid (3 mM; MeAIB; M2383; Sigma Aldrich).

Guest cells (MiaPaCa-2 wt, xCT^KO^ or xCT-ASCT2^KO^) were also cultivated in full conditional media (CM) of HDF wt, LS174T wt, A549 wt, Capan-2 wt, Capan-2 GCLc^KO^, Capan-2 xCT^KO^ cells for 24 h or 48 h, as indicated. The CM were obtained by seeding 1 × 10^6^ cells in 10 cm dishes containing 10 mL of DMEM + 7.5% FBS at 37 °C/5% CO_2_ for 24 h, after this period the media was collected and centrifuged for 10 min at 1000 rpm and immediately used in the subsequent assays. As indicated in the Results section, CM of HDF was centrifuged for fractionalization with 3 kDa cut-off filters (Amicon^®^ ultra-15 centrifugal filter units, Millipore) or dialyzed against PBS buffer 1× using a 3 kDa cut-off membrane (Spectrum Laboratories, Inc. Waltham, MA, USA).

### 5.5. FACS Analysis

FACS analysis of cell death and lipid hydroperoxides accumulation were performed as described by Daher et al. [10]. Experiments were performed at least 3 times; 10 000 events were obtained per sample using BD FACSMelody cytometer and data were analyzed using FlowJo software (Ashland, USA).

Cell death: Cells were seeded in 6-well dishes and collect, together with the corresponding supernatant, after 48 h. Following centrifugation, the cells were re-suspended in FACS buffer (PBS, 0.2% BSA, 2 mM EDTA) and stained, just before the analysis, with 2 μg/mL PI (Invitrogen, Waltham, MA, USA).

Detection of lipid hydroperoxides: The cells were seeded in 6-well dishes and in the day of analysis, incubated with 2 μM BODIPY 581/591 C11 (Molecular Probes, Eugene, OR, USA) for 30 min at 37 °C/5% CO_2_ protected from the light. Following, the cells were washed 2 times with PBS 1×, collected with accutase (Dutscher, Bernolsheim, France) and resuspended in FACS buffer (PBS, 0.2% BSA, 2 mM EDTA). The data are represented in modal scaling (each peak is normalized to its mode, i.e., to % of maximal number of cells found in a particular bin).

### 5.6. Immunoblotting

Cells were lysed in 1.5× Laemmli buffer, and protein concentrations were determined using BCA protein assay (ThermoFisher Scientific, Waltham, MA, USA). 20–40 μg of total protein extracts were separated by electrophoresis in 10% SDS-PAGE and transferred onto PVDF membranes (Immobilon, Merck Millipore Ltd., Tullagreen, Carrigtwohill, Co. Cork, Ireland). The membranes were blocked with 5% non-fat milk in PBS and incubated with anti-human primary antibodies against: xCT (1/1000; 12691; Cell Signaling Technology—CST, Leiden, Netherlands), phospho-GCN2 (1:500; ab75836; Abcam, Cambridge, UK), rabbit ATF4 (1:1000; 11815S; CST), phospho-S6RP (1:1000; 2215S, CST); phospho-S6K (1:1000; 9202S; CST), LAT1 (SLC7A5; 1:1000, KE026, TransGenic Inc., Illkirch-Graffenstaden, France), ASCT1 (SLC1A4; 1:1000; 84424; CST), ASCT2 (SLC1A5; 1:1000; 8057S, CST), EAAT3 (1:1000; ab124802, Abcam), EAAT4 (1:1000, ab186435, Abcam) or CHAC1 (1 μg/mL; AV42623; Sigma Aldrich). Protein loading control was verified by detection of tubulin (1:1000; MA5-16308; Thermo Scientific, Waltham, MA, USA) or ARD1 (rabbit anti-human/mouse; homemade [42]). Immunoreactive bands were detected with horseradish peroxidase antimouse or antirabbit antibodies (Promega, Medison, WI, USA) using ECL system (Merck Milipore, Watford, UK). Immunoblot analysis was performed using the LI-COR Odyssey Imaging System (Lincoln, NE, USA).

### 5.7. Uptake of Radioactive-Labeled Cysteine

The influence of ASCTs, SNATs, and LAT1 transporters on the uptake of ^14^C-cysteine was measured. Cells (2.5 × 10^5^) were seeded into 35 mm dishes and after 24 h, culture media were removed and cells were washed with Hank’s Balanced Salt Solution (HBSS: 125 mM NaCl, 4.8 mM KCl, 1.2 mM MgSO_4_, 1.2 mM NaH_2_PO_4_, 1.3 mM CaCl_2_, 5.6 mM glucose, and 25 mM HEPES), incubated in 1 mL of prewarmed HBSS at 37 °C for 5 min. Following, the cells were incubate at room temperature for 30 min in 1 mL HBSS supplemented with L-[1,2,1′,2′-^14^C]cysteine (0.2μCie/mL; PerkinElmer Ref: NEC854010UC) + 100 μM β-mercaptoethanol, in order to keep cysteine in its reduced form) and 50 μM cold cystine in the presence or not of 10 mM L-alanine (ASCTs transporters competitive inhibitor), 10 mM L-Leucine (LAT1 transporter competitive inhibitor) or 10 mM N-methylaminoisobutyric acid (MeAIB, Merck, Darmstadt, Germany, SNATs transporters inhibitor). Subsequently, cells were washed 3 times with HBSS solution containing the specific carriers’ inhibitors and lysed with 1 mL of 1 M NaOH, following addition of 12 mL of Emulsifier-Safe cocktail (PerkinElmer, Waltham, MA, USA). Radioactivity was measured using a β–scintillation counter. Relative L-[^14^C-cysteine] uptake was normalized by protein content. L-alanine (A7627), L-leucine (L8000), and MeAIB (M2383) were purchased from Sigma-Aldrich.

### 5.8. Statistical Analysis

Data are expressed as mean ± SEM. Each experiment was performed at least three times. Statistical analysis was done with the unpaired Student t test. Differences between groups were considered statistically significant when *p* < 0.05.

## Figures and Tables

**Table 1:** Summary of the different combinations of the mutations in the “host” and “guest” cells and their effect on the lipid hydroperoxide profile and cell death.

GUEST/HOST	A549 wt	A549 ASCT1^KO^	A549 ASCT2^KO^	A549 ASCT1-ASCT2^DKO^	A549 LAT1^KO^
MiaPaCa-2 wt	= ✓	= ✓	= ✓	= ✓	= ✓
MiaPaCa-2 xCT^KO^	= ✓	= ✓	↑ ✓	↑ ✗	= ✓
MiaPaCa-2 xCT-ASCT2^DKO^	= ✓	= ✓	↑ ✗	↑ ✗	= ✓

* **=**, no change in the lipid hydroperoxide profile; **↑**, increase in the lipid hydroperoxide content; **✓**, no cell death; ✗, cell death observed.

## Data Availability

Data is contained within the article or Supplementary Material. The data presented in this study are available in www.mdpi.com/xxx/s.

## Supplementary Materials

The following are available online at https://www.mdpi.com/2072-6694/13/6/1434/s1, Figure S1: Redox nature of the “saving agent; Figure S2: Cell-to-cell interplay is fueled by the xCT activity of the host cells; Figure S3: xCTKO and GClcKO cells as hosts; Figure S4: Potential cysteine transporters; Figure S5: The involvement of cysteine transporters in cysteine-cystine shuttle; Figure S6: Pharmacological inhibition of cysteine transporters.
