# Supplementary material for: A Cystine-Cysteine Intercellular Shuttle Prevents Ferroptosis in xCTKO Pancreatic Ductal Adenocarcinoma Cells
Source: Cancers (Basel). 2021 Mar 21;13(6):1434. doi: 10.3390/cancers13061434 (PMC8004104; doi:10.3390/cancers13061434)
Supplement: Supplementary file 1 [file cancers-13-01434-s001.pdf]

SUPPLEMENTARY FIGURE LEGENDS:

Figure S1. **Redox nature of the "saving agent" A.** Micrographs of MiaPaCa-2 xCT^{KO} cells in control conditions co-culture with LS174T wt in DMEM or DMEM supplemented with glucose oxidase (0,2mU/ml GOx), **B.** ROS level in MiaPaCa-2 wt and two independent xCT^{KO} clones in control conditions, after addition of NAC or in the co-culture (CC) with LS174T/A549 wt cells for 24h. The cells were seeded in 6-well dishes and in the day of analysis, trypsinized and incubated with 2μM DCFDA (Abcam) for 30 minutes at 37ºC/5% CO_2 protected from the light. Following, FACS analysis of total ROS level were performed and the data are represented in modal scaling (each peak is normalized to its mode, i.e., to % of maximal number of cells found in a particular bin). Representative histograms are shown, and the bar graph represent mean ± SEM; n=3; *, P<0.05, comparison with WT control of each group.

Figure S2. **Cell-to-cell interplay is fueled by the xCT activity of the host cells. A.** The effects of buthionine sulphoximine (BSO) and erastin on the lipid peroxide accumulation in the A549 wt, measured by BODIPY 591/581 C11 staining. **B.** The effects of media containing or not cyst(e)ine on the BODIPY 591/581 C11 staining in the A549 wt alone or in the co-culture with MiaPaCa-2 xCT^{KO} cells. Representative histograms of three independent experiments are shown.

Figure S3. **xCTKO and GClcKO cells as hosts.** Proliferation rate of the Capan-2 wt, xCT^{KO} and $GCLc^{KO}$ cells does not correlate with their ability to prevent ferroptosis in MiaPaCa-2 xCT^{KO} counterpart. **A.** Proliferation rates of the Capan-2 wt, xCT^{KO} and $GCLc^{KO}$ cells are presented as fold of change (mean ± SEM; n = 2). **B.** Proliferation rate (48h), **C.** lipid hydroperoxide accumulation (24h) and **D.** visualization (48h) of MiaPaCa-2 wt and two independent xCT^{KO} clones cultivated in the presence or not of NAC, Capan-2 wt, xCT^{KO} and $GCLc^{KO}$ (Boyden chamber, mean ± SEM; n = 2). The host cells were pre-seeded in the upper well of the Boyden chamber (Falcon® Permeable Support for 24-well Plate with 8.0 μm Transparent PET Membrane), and 24h later, guest cells (MiaPaCa-2 wt or xCTKO) were added in the lower well (the same 6%/94% ratio has been maintained for the experiment as in the case of the co-culture). After 24/48h upper well had been removed and the guest cells from the lower well were analyzed for lipid hydroperoxide accumulation (FACS analysis, 24h), proliferation (48h), or cell survival (Giemsa staining, 48h).

Figure S4: **Potential cysteine transporters. A.** ASCT2 expression was analysed in the clones obtained after transfection of ASCT2 CRISPR-Cas9 plasmide in the MiaPaCa-2 xCT^{KO} cells and two independent xCT-$ASCT2^{DKO}$ are chosen for further experiments (cl.1 and cl.2). **B.** The expression levels of ASCT1, ASCT2 and LAT1 in A549: wt, $ASCT1^{KO}$, $ASCT2^{KO}$, ASCT1-$ASCT2^{DKO}$, $LAT1^{KO}$ after 24h culturing in the DMEM, ARD1 were used as loading control. Representative blots are shown. **C.** Clonal growth

of A549: wt, ASCT1^{KO}, ASCT2^{KO}, ASCT1-ASCT2^{DKO} and LAT1^{KO}. Cells (10^3) were cultivated for 15 days in regular DMEM media or DMEM media supplemented with 200μM CySSCy, 400μM CySH or 10mM MeAIB (inhibitor of SNAT transporters family) and colored for visualizations with Giemsa. Representative images are shown. #NB, First row represents regular DMEM supplemented with 8% FBS, while in all other cases media lacking cyst(e)ine and supplemented with 10% dialysed FBS (dFBS) has been used and the CySSCy/CySH/MeAIB adjusted thereafter.

Figure S5: The involvement of cysteine transporters in cysteine-cystine shuttle. A. Proliferation rate of A549 wt, ASCT1^{KO}, ASCT2^{KO}, ASCT1-ASCT2^{DKO} and LAT1^{KO} cells for 3 days; presented as fold of change (mean ± SEM; n = 2). **B.** Cell death of MiaPaCa-2 wt, xCT^{KO} and xCT-ASCT2^{DKO} in control conditions, in the presence of NAC or cultivated in the presence of A549 wt (left panel) or ASCT1-ASCT2^{DKO} (right panel) cells (Boyden chamber). The bar graph represent mean ± SEM; n=2; *, $P<0.05$, comparison with WT control of each group.

Figure S6: **Pharmacological inhibition of cysteine transporters.** Cell viability of the MiaPaCa-2 wt, xCT^{KO} or xCT-ASCT2^{DKO} co-cultured with A549 wt cells in the presence or not of 1mM NAC, 3mM L-alanine, 3mM L-leucine or 3mM MeAIB after 48h. Bar graph shows mean ± SEM; n=3; *, $P<0.05$, comparison with WT control of each group.

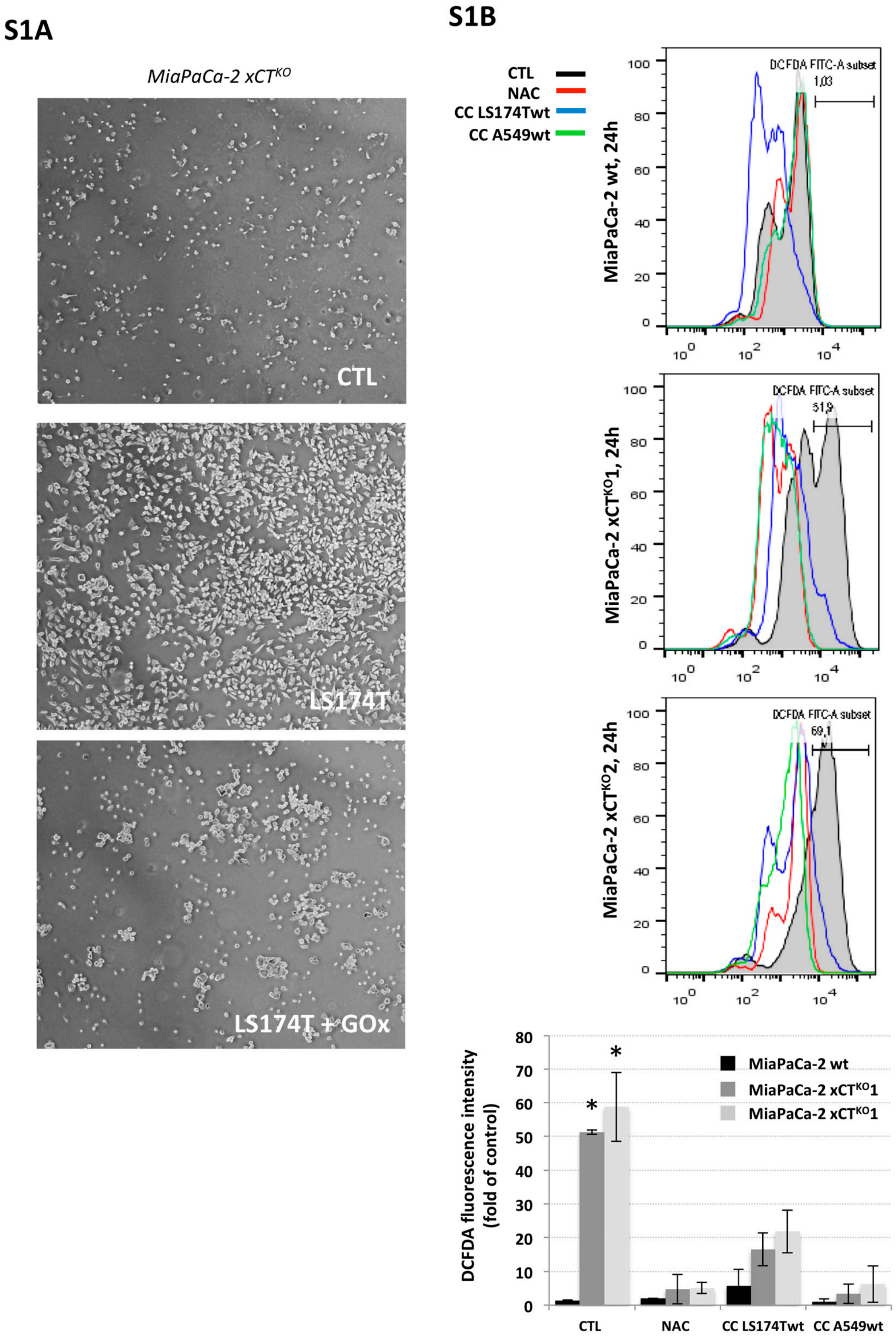
S1A
MiaPaCa-2 xCTKO
CTL
LS174T
LS174T + GOx
S1B
CTL
NAC
CC LS174Twt
CC A549wt
MiaPaCa-2 wt, 24h
DCFDA FITC-A subset
1,03
MiaPaCa-2 xCTKO1, 24h
DCFDA FITC-A subset
51,9
MiaPaCa-2 xCTKO2, 24h
DCFDA FITC-A subset
69,1
DCFDA fluorescence intensity (fold of control)
MiaPaCa-2 wt
MiaPaCa-2 xCTKO1
MiaPaCa-2 xCTKO1
CTL
NAC
CC LS174Twt
CC A549wt

Figure S1

S2A

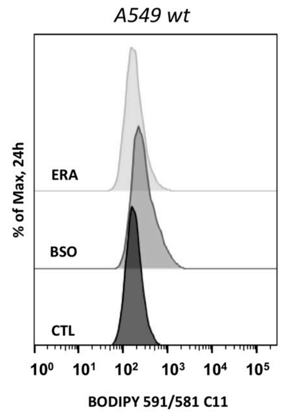

S2B

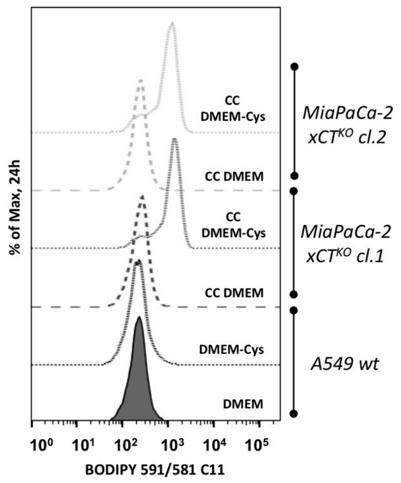

Figure S2

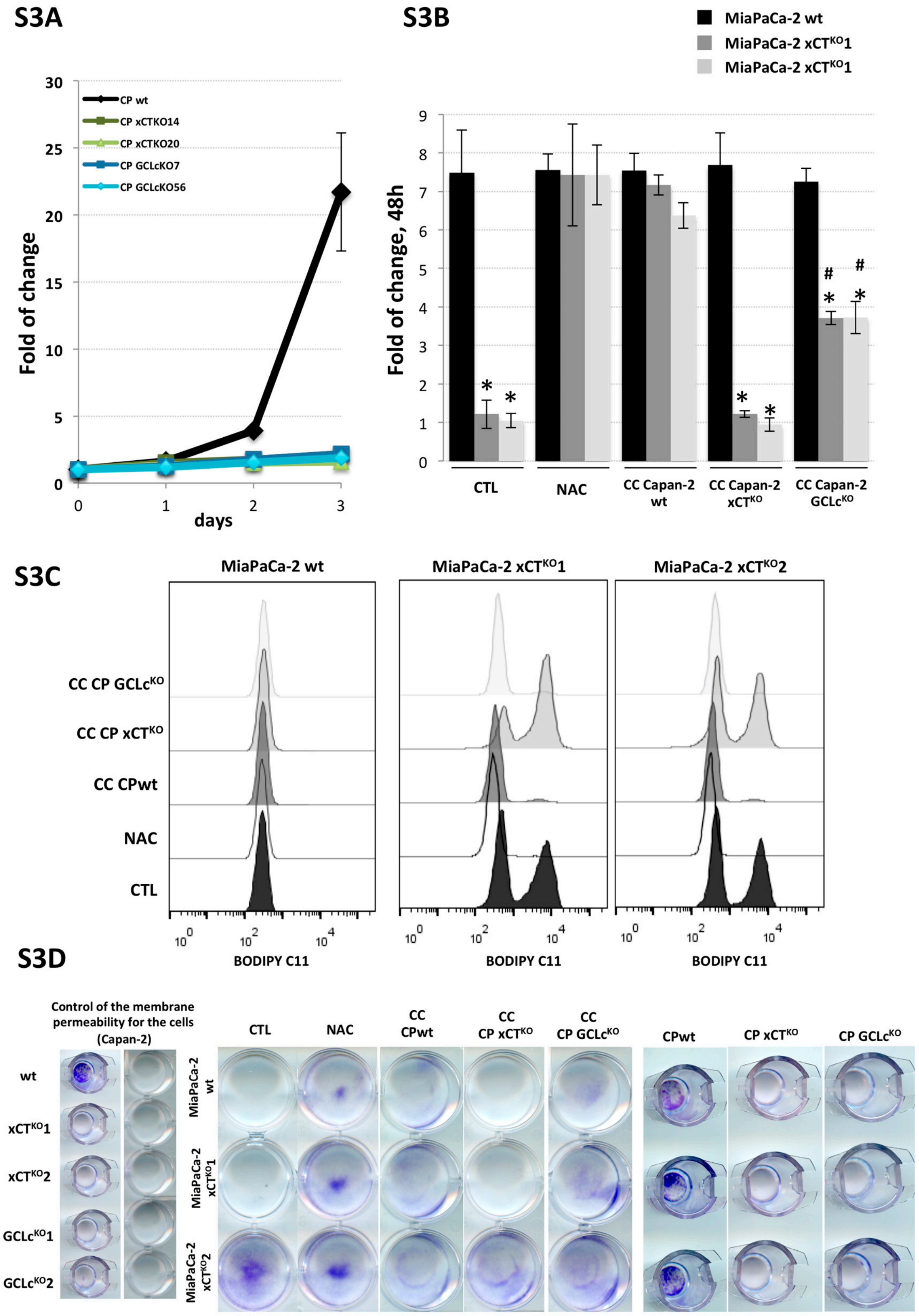
S3A
CP wt
CP xCTKO14
CP xCTKO20
CP GCLcKO7
CP GCLcKO56
Fold of change
days
S3B
MiaPaCa-2 wt
MiaPaCa-2 xCT^KO1
MiaPaCa-2 xCT^KO1
Fold of change, 48h
CTL
NAC
CC Capan-2 wt
CC Capan-2 xCT^KO
CC Capan-2 GCLc^KO
S3C
MiaPaCa-2 wt
MiaPaCa-2 xCT^KO1
MiaPaCa-2 xCT^KO2
CC CP GCLc^KO
CC CP xCT^KO
CC CPwt
NAC
CTL
BODIPY C11
S3D
Control of the membrane permeability for the cells (Capan-2)
wt
xCT^KO1
xCT^KO2
GCLc^KO1
GCLc^KO2
MiaPaCa-2 wt
MiaPaCa-2 xCT^KO1
MiaPaCa-2 xCT^KO2
CTL
NAC
CC CPwt
CC CP xCT^KO
CC CP GCLc^KO
CPwt
CP xCT^KO
CP GCLc^KO

Figure S3

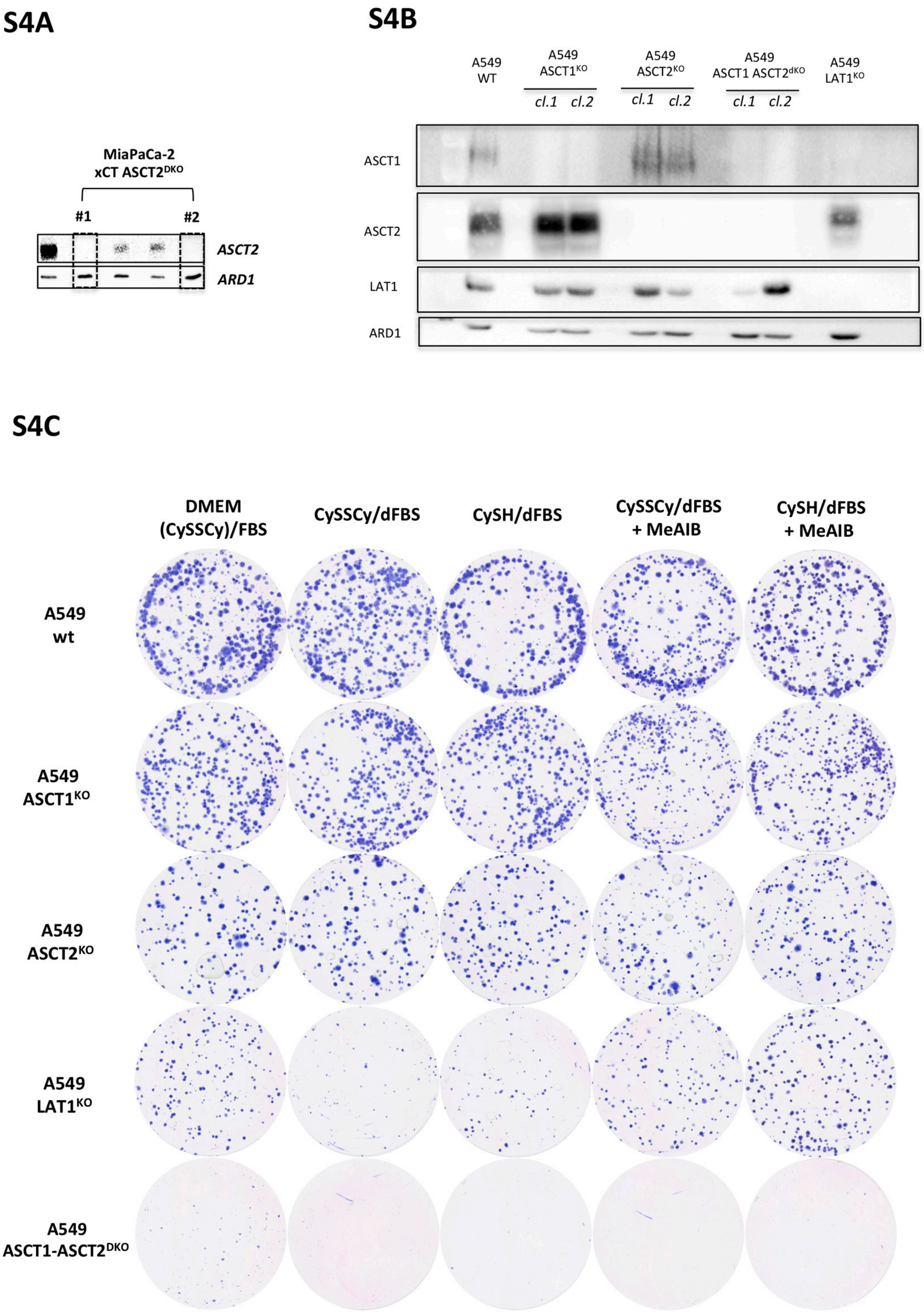
S4A
MiaPaCa-2
xCT $ASCT2^{DKO}$
#1
#2
ASCT2
ARD1
S4B
A549 WT
A549 $ASCT1^{KO}$
A549 $ASCT2^{KO}$
A549 ASCT1 $ASCT2^{dKO}$
A549 $LAT1^{KO}$
cl.1
cl.2
cl.1
cl.2
cl.1
cl.2
ASCT1
ASCT2
LAT1
ARD1
S4C
DMEM (CySSCy)/FBS
CySSCy/dFBS
CySH/dFBS
CySSCy/dFBS + MeAIB
CySH/dFBS + MeAIB
A549 wt
A549 $ASCT1^{KO}$
A549 $ASCT2^{KO}$
A549 $LAT1^{KO}$
A549 ASCT1-$ASCT2^{DKO}$

Figure S4

S6

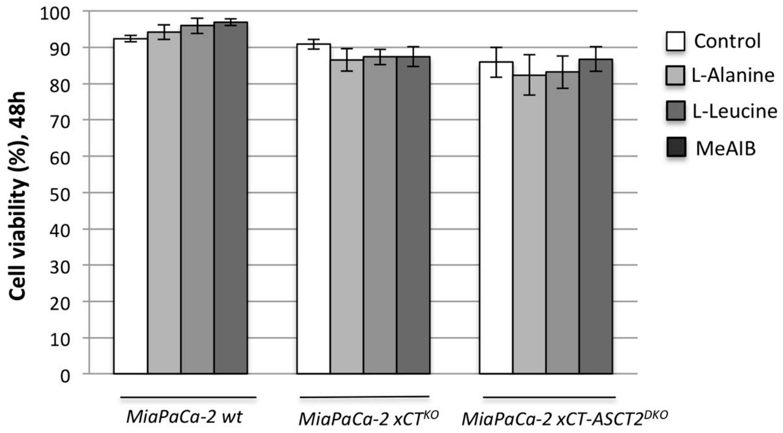
100
90
80
70
60
50
40
30
20
10
0
Cell viability (%), 48h
Control
L-Alanine
L-Leucine
MeAIB
MiaPaCa-2 wt
MiaPaCa-2 xCT^KO
MiaPaCa-2 xCT-ASCT2^DKO

Figure S5

Figure S6

Additional supplementary data

Add Supp for Fig 1B Example of the FSC/SSC plots for the co-culture after 24h – « guest » and « host » cells are undistinguishable

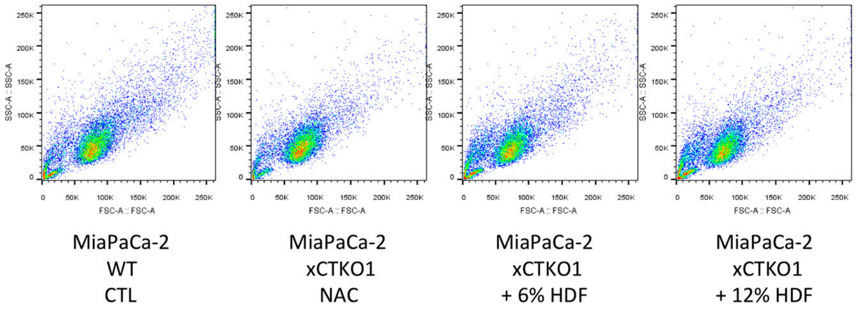

Add Supp for Fig 3C Accumulation of lipid hydroperoxides MiaPaCa-2 xCT^{KO} cells (two independent clones) in co-culture (CC) or cultivated in the presence of conditional media (CM) of Capan-2 $GCLc^{KO}$ or xCT^{KO} cells (**two independent clones for each cell line**) during 24h. Representative histogram of three independent experiments are shown

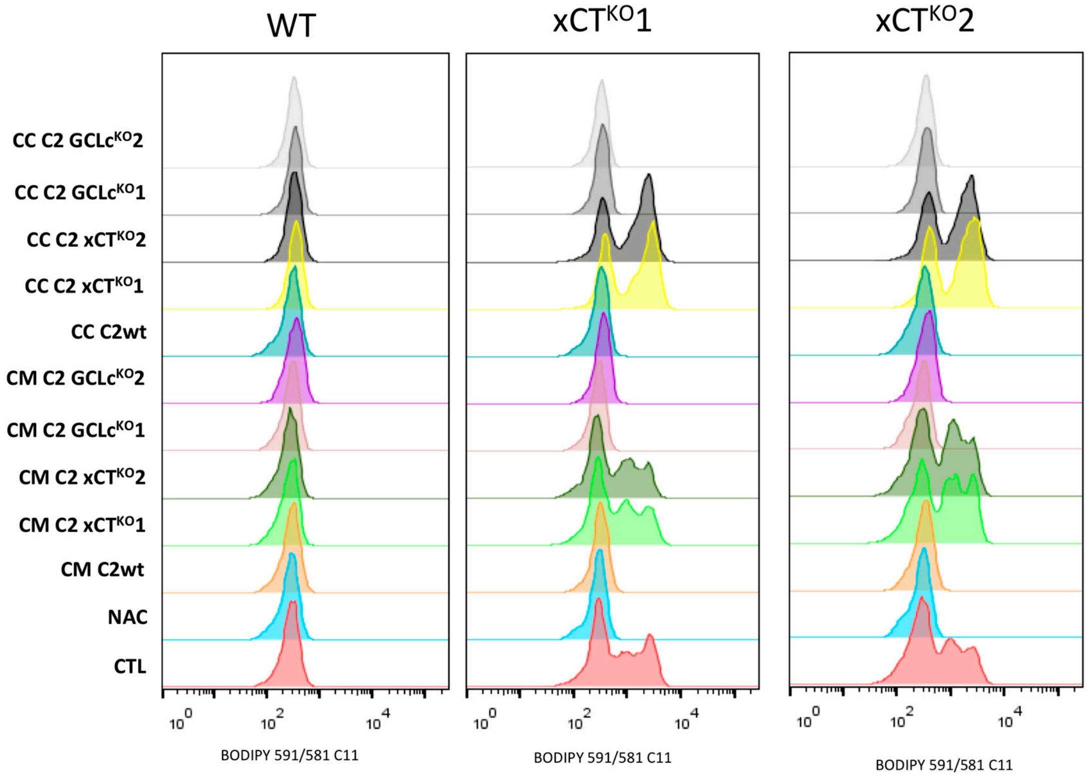

Add Supp for Fig 4B Lipid hydroperoxide accumulation of MiaPaCa-2 xCT^{KO} and xCT-ASCT2^{DKO} cells (guest cells- CySH import) in control conditions or co-cultured with 6% A549 wt, ASCT1^{KO}, ASCT2^{KO} or ASCT1-ASCT2^{DKO} (host cells- CySH export). Representative histogram (n=3) of additional, independent clone for each KO/DKO-cell line.

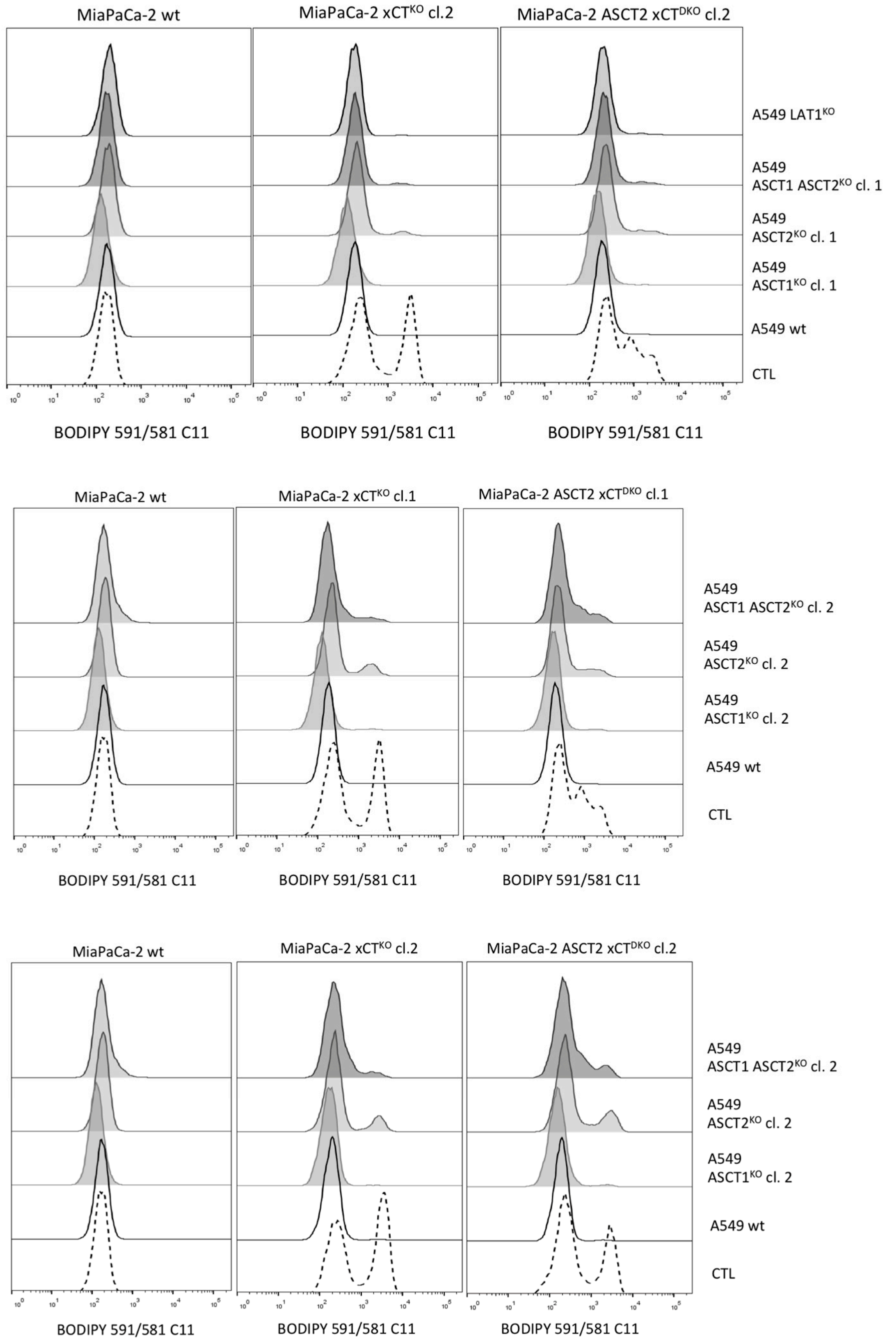

Add Supp for Fig 4B Cell viability of MiaPaCa-2 xCT^{KO} and $xCT\text{-}ASCT2^{DKO}$ cells (guest cells- CySH import) in control conditions or co-cultured with 6% A549 wt, $ASCT1^{KO}$, $ASCT2^{KO}$ or $ASCT1\text{-}ASCT2^{DKO}$ (host cells- CySH export). Bar graph shows mean ± SEM; n=3; *, P<0.05, comparison with the corresponding control group. Data for two independent clones for each KO/DKO-cell line.

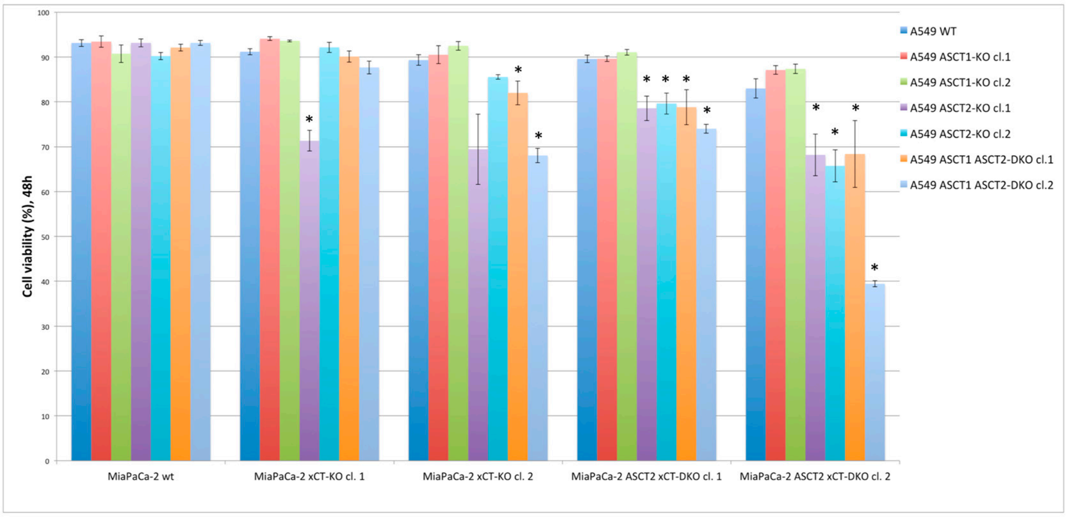

Add Supp for Fig 5B Accumulation of the lipid hydroperoxides in MiaPaCa-2 wt, xCT^{KO} or xCT-$ASCT2^{DKO}$ (guest cells) co-cultured with A549 wt cells (host cells) in the presence or not of the 1mM NAC, 3mM L-alanine, 3mM L-leucine or 3mM MeAIB after 24h. Representative histogram (n=3) of additional, independent clone for each KO/DKO-cell line.

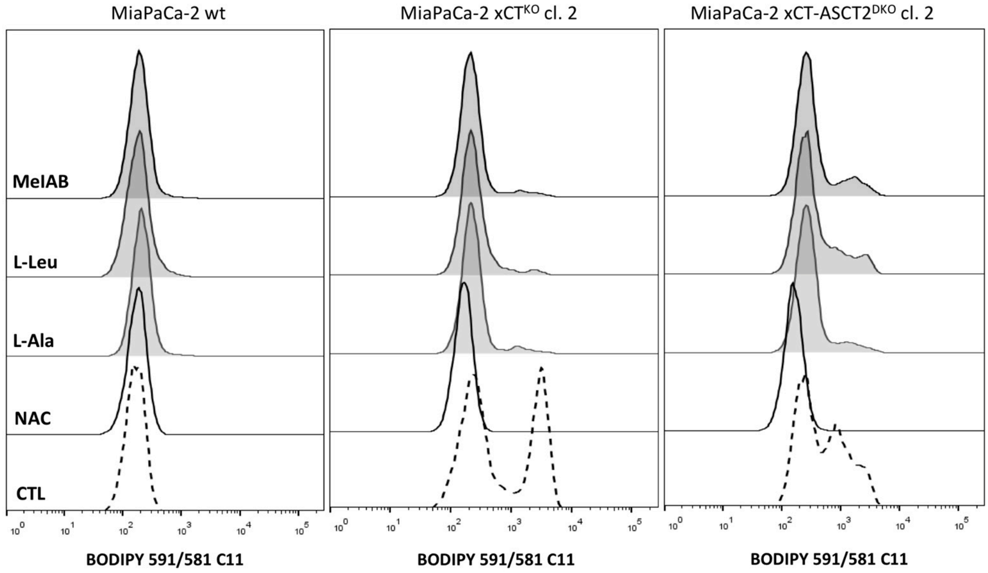

Add Supp for Fig S4 Cell viability of the MiaPaCa-2 wt, xCT^{KO} or xCT-$ASCT2^{DKO}$ co-cultured with A549 wt cells in the presence or not of 1mM NAC, 3mM L-alanine, 3mM L-leucine or 3mM MeAIB after 48h. Bar graph shows mean ± SEM; n=3; *, P<0.05, comparison with WT control of each group. Data for additional, independent clone for each KO/DKO-cell line.

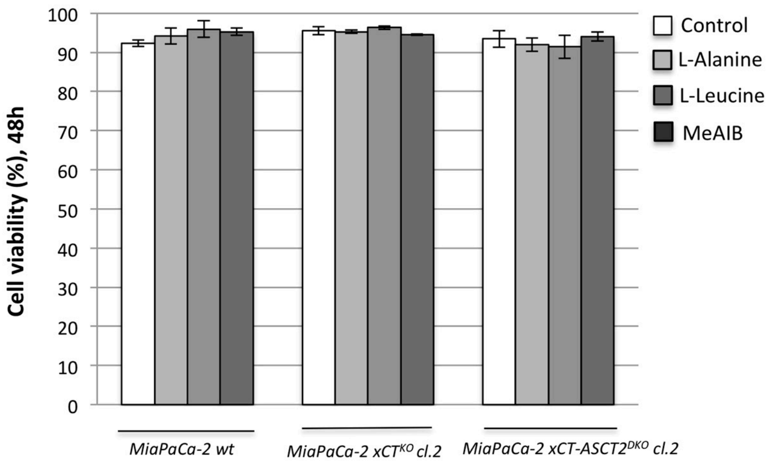

Add Supp for Fig 1A, 2D Original WB membranes

1A

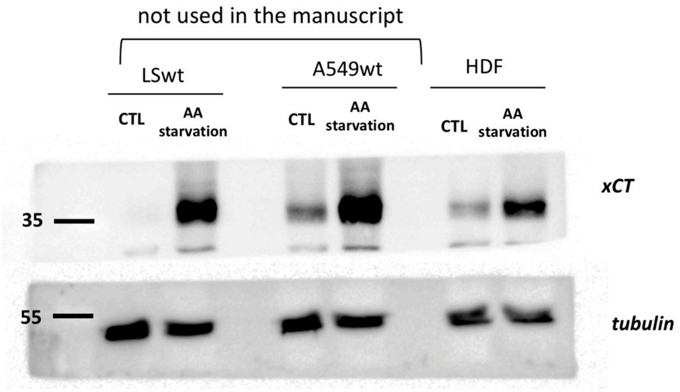

2D

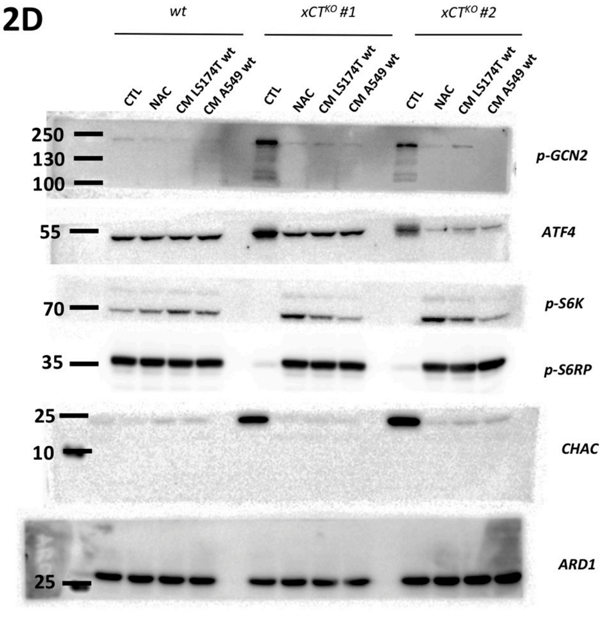

Add Supp for Fig 4A Original WB membranes

4A

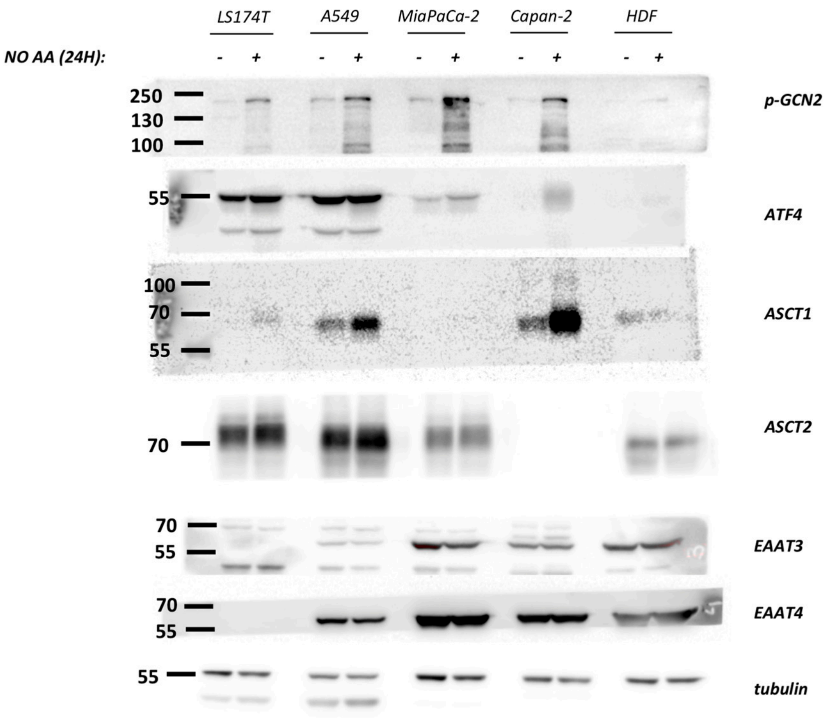

Add Supp for Fig S3A, S3B Original WB membranes

Supp3A

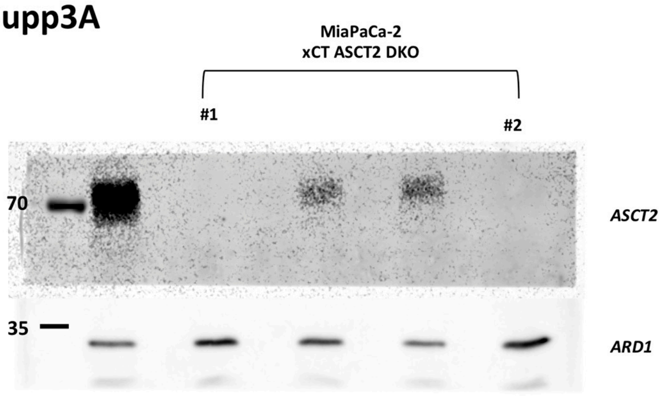

Supp3B

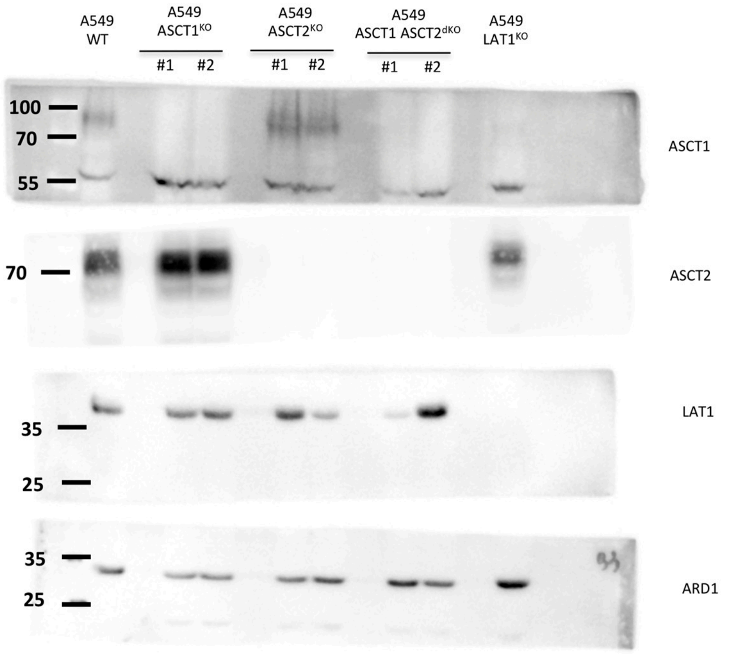

Add Supp for Fig 1A, 2D, 4A, S3A, S3B Densitometry readings for WB blots

Fig. 1A		
	CTL	AA starvation
xCT	7137.116	19047.350
tubuline	19430.208	18175.865

Fig. 2D												
	wt				xCTKO #1				xCTKO #2			
	CTL	NAC	CM LS174T wt	CM A549 wt	CTL	NAC	CM LS174T wt	CM A549 wt	CTL	NAC	CM LS174T wt	CM A549 wt
p-GCN2	751.820	572.749	426.335	269.335	5883.045	603.406	543.698	297.506	4440.296	331.920	591.042	0
ATF4	6981.539	6826.660	6729.146	8109.924	15347.794	7852.045	8522.803	7641.146	12167.279	2319.104	3711.903	3201.832
p-S6K	2342.083	4000.225	5264.933	3964.861	128.021	7044.347	5179.225	2325.548	267.263	7995.296	5777.690	2850.983
p-S6RP	14075.823	14924.359	14420.187	14256.702	1964.912	14976.480	14802.187	14202.652	1326.012	12528.823	15354.844	17168.673
CHAC	1113.184	1028.648	1260.305	1546.912	12688.803	2026.497	1722.962	1321.184	16192.116	2363.397	1880.033	1337.376
tubuline	8321.196	9808.217	9858.095	9068.267	7739.317	8347.317	7898.853	7258.903	9389.803	8793.489	8268.075	5961.054

Fig. 4A										
	LS174T		A549		MiaPaCa-2		Capan-2		HDF	
No AA (24h)	−	+	−	+	−	+	−	+	−	+
p-GCN2	462.335	1732.376	1313.134	2907.983	3053.468	7354.823	1005.062	2601.790	0	260.799
ATF4	10107.288	12070.844	13426.016	13442.551	1390.033	1813.861	0	3950.033	0	247.192
ASCT1	323.799	362.920	11461.309	14665.602	0	0	17804.693	27347.836	3287.276	2268.376
ASCT2	11999.572	13561.421	15493.078	18526.635	9102.823	8967.995	0	0	3529.510	4279.874
EAAT3	223.021	153.778	1208.062	1033.234	8786.468	8735.175	3848.983	3992.640	7017.004	7417.368
EAAT4	0	0	6194.660	5649.418	12520.146	13011.803	9925.560	8980.075	5782.660	7813.953
tubuline	4533.376	4253.790	3359.790	3142.376	7274.518	5887.690	5030.104	4270.154	5239.276	5164.569

Supplementary 3A					
MiaPaCa-2 xCT ASCT2 DKO					
		#1			#2
ASCT2	34884.685	0	5720.359	6686.409	0
ARD1	5967.640	8798.125	6970.761	4414.276	10329.903

Supplementary Fig. 3B								
	A549 WT	A549 ASCT1KO #1	A549 ASCT1KO #2	A549 ASCT2KO #1	A549 ASCT2KO #2	A549 ASCT1-ASCT2KO #1	A549 ASCT1-ASCT2KO #2	A549 LAT1KO
ASCT1	7596.572	11640.350	11908.693	0	0	0	0	494.678
ASCT2	11372.773	19162.836	17526.208	0	0	0	0	7060.752
LAT1	6412.560	5812.782	7520.095	8592.267	3204.296	1137.891	12201.752	0
ARD1	3939.276	2717.619	3342.861	3502.154	4983.104	6155.761	4155.861	8386.539
